# Pharmacological AMPK activation induces transcriptional responses congruent to exercise in skeletal and cardiac muscle, adipose tissues and liver

**DOI:** 10.1371/journal.pone.0211568

**Published:** 2019-02-27

**Authors:** Eric S. Muise, Hong-Ping Guan, Jinqi Liu, Andrea R. Nawrocki, Xiaodong Yang, Chuanlin Wang, Carlos G. Rodríguez, Dan Zhou, Judith N. Gorski, Marc M. Kurtz, Danqing Feng, Kenneth J. Leavitt, Lan Wei, Robert R. Wilkening, James M. Apgar, Shiyao Xu, Ku Lu, Wen Feng, Ying Li, Huaibing He, Stephen F. Previs, Xiaolan Shen, Margaret van Heek, Sandra C. Souza, Mark J. Rosenbach, Tesfaye Biftu, Mark D. Erion, David E. Kelley, Daniel M. Kemp, Robert W. Myers, Iyassu K. Sebhat

**Affiliations:** 1 Genetics and Pharmacogenomics Department, MRL, Kenilworth, NJ, United States of America; 2 Biology-Discovery Department, MRL, Kenilworth, NJ, United States of America; 3 In Vivo Pharmacology Department, MRL, Kenilworth, NJ, United States of America; 4 In Vitro PharmacologyDepartment, MRL, NJ, United States of America; 5 Medicinal ChemistryDepartment, MRL, Kenilworth, NJ, United States of America; 6 PPDM Preclinical ADME Department, MRL, Kenilworth, NJ, United States of America; 7 SALAR Department, MRL, Kenilworth, NJ, United States of America; Université catholique de Louvain, BELGIUM

## Abstract

Physical activity promotes metabolic and cardiovascular health benefits that derive in part from the transcriptional responses to exercise that occur within skeletal muscle and other organs. There is interest in discovering a pharmacologic exercise mimetic that could imbue wellness and alleviate disease burden. However, the molecular physiology by which exercise signals the transcriptional response is highly complex, making it challenging to identify a single target for pharmacological mimicry. The current studies evaluated the transcriptome responses in skeletal muscle, heart, liver, and white and brown adipose to novel small molecule activators of AMPK (pan-activators for all AMPK isoforms) compared to that of exercise. A striking level of congruence between exercise and pharmacological AMPK activation was observed across the induced transcriptome of these five tissues. However, differences in acute metabolic response between exercise and pharmacologic AMPK activation were observed, notably for acute glycogen balances and related to the energy expenditure induced by exercise but not pharmacologic AMPK activation. Nevertheless, intervention with repeated daily administration of short-acting activation of AMPK was found to mitigate hyperglycemia and hyperinsulinemia in four rodent models of metabolic disease and without the cardiac glycogen accretion noted with sustained pharmacologic AMPK activation. These findings affirm that activation of AMPK is a key node governing exercise mediated transcription and is an attractive target as an exercise mimetic.

## Introduction

Physical activity contributes to wide-ranging health benefits that include prevention or delay in the progression of metabolic disorders including insulin resistance, obesity, type 2 diabetes mellitus (T2DM) and cardiovascular disease [1]. The health promoting effects of exercise can be categorized as deriving partly from relatively transient effects of substrate utilization that occurs during exercise and partly from induction of tissue plasticity, mediated by exercise-induced transcriptional effects. Transcriptional changes evoked by exercise have been characterized for skeletal muscle [2, 3], though these effects in other organs are less fully described [4]. A recent study of phosphoproteome induced acutely in response to exercise in skeletal muscle, indicates the network of signaling pathways is highly complex [5]. This comprehensive effort identified activation of pathways earlier reported to be stimulated by exercise, including 5'-adenosine monophosphate activated protein kinase (AMPK), calcium/calmodulin-dependent kinases, calcineurin, mitogen-activated protein kinase and mammalian target of rapamycin. However, the phosphorylation of peptides attributable to these pathways appeared to account for just 10% of the exercise-evoked phosphoproteome indicating contribution, indeed major participation, from signaling pathways yet to be characterized.

There are many individuals burdened with metabolic diseases, such as type 2 diabetes mellitus, who due to the complications of this illness and related co-morbidities are unable to undertake exercise, or at least of sufficient duration or intensity. Pharmacological approaches that could recapitulate effects of exercise could potentially have considerable benefit for these individuals [6]. For practical reasons, pharmacology most often focuses upon a single molecular target and accordingly, a central question that arises with respect to the complex panoply of signaling responses evoked by exercise [5], is how effectively can a single target pharmacology recapitulate the effects of exercise. Potential targets that might be employed as an exercise mimetic and respective mechanisms and rationale have recently been reviewed [6] and these are numerous, including ligands to activate AMPK, PPAR δ, REV-ERBα, sirtuin 1, ERRα and others. In the studies reported herein, we tested the hypothesis that pharmacological activation of AMPK could serve as an exercise mimetic with respect to governance of the transcriptional response.

Activation of AMPK has long been regarded as one of the crucial signaling nodes responsible for the transcriptional response to exercise [7]. In human and rodent skeletal muscle, AMPK is acutely activated via phosphorylation in response to exercise [8–12], though this effect is less robust or absent at low-intensity physical activity. AMPK is a heterotrimer comprised of catalytic α (2 isoforms), "scaffold" β- (2 isoforms), and regulatory γ- (3 isoforms) subunits [13]. Mammals can express up to 12 different isoform combinations and distribution of these is tissue- and species-specific [14]. Our group recently reported the improvement of hyperglycemia in animal models of T2DM using a long-acting AMPK activator, MK-8722 [15], and similar preclinical efficacy of a structurally related series of small molecule AMPK activators has also been recently described [16]. Both reports emphasized that sustained engagement of the AMPK β_2_-containing isoforms in skeletal muscle and a sustained downstream effect to stimulate glucose transport into muscle is the key mechanism for the glucose lowering efficacy of these agents. However, sustained stimulation of glucose transport was also observed in cardiac muscle and administration of a long-acting AMPK activator caused increased heart weight and glycogen deposition [15], which raised important safety concerns limiting prospects for drug development of long-acting AMPK activators.

The current studies were undertaken primarily to explore whether pharmacological AMPK activation might mimic exercise in its transcriptional response. The transcriptional response to pharmacological AMPK activation was extensively profiled across five tissues simultaneously (skeletal muscle, heart, liver, white and brown adipose) and compared to the response to exercise. A second goal was to employ short-acting versus long-acting AMPK activators. A short-duration AMPK activator is more akin to the duration of a bout of physical activity than sustained pharmacological AMPK activation and though the resultant stimulation of glucose transport into skeletal muscle is also correspondingly short-lived, we tested the hypothesis that daily administration of short-acting AMPK activators due to its transcriptional response may have a disease modifying effect on diabetes and insulin resistance.

## Results

### Discovery of potent, specific, pan-activators of mammalian AMPK

**Fig 1:**
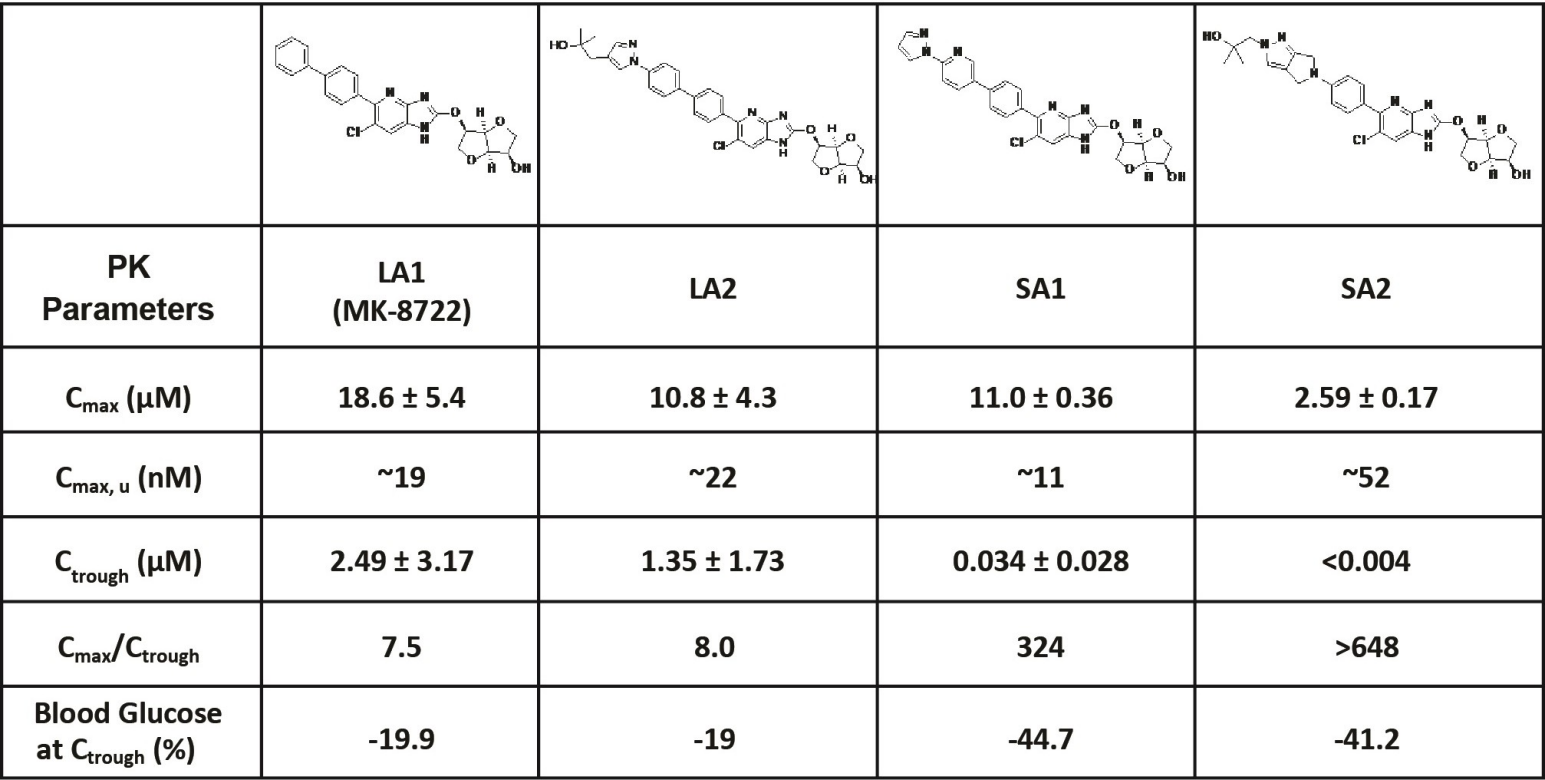
Structures and pharmacokinetic parameters of LA1, LA2, SA1 and SA2. Pharmacokinetic studies were performed in 8 week old db/db mice treated with LA1 (10 mg/kg), LA2 (30 mg/kg), SA1 (30 mg/kg), SA2 (20 mg/kg) for 14 days (QD, PO). Blood samples were collected via tail vein at 0, 1, 2, 4, 7, and 24h post dosing on day 14 (n = 8). C_max, u_ is the unbound C_max_ (when accounting for plasma protein binding). Blood Glucose at C_trough_ (%) is the percent change of blood glucose of mice treated with different compounds compared to vehicle at 24h post dose on day 14. Data are represented as mean ± SEM.

The medicinal chemistry effort to create a series of novel compounds with potent activities against all twelve isoforms of AMPK complexes has been previously described [15]. These compounds exhibited EC50 values of ~1–34 nM, have excellent cell permeability, and achieve >200% activation of AMPK relative to the maximal activation induced by AMP (S1 Table). Pharmacokinetic (PK) studies demonstrated that the compounds are orally bioavailable and achieve similar unbound peak plasma drug concentration (C_max, u_) but differ substantially in respective durations of action. In the current studies, four AMPK activators were used, two long-acting compounds (LA1, LA2) and two short-acting compounds (SA1, SA2), the structure of each is shown in Fig 1. LA1 is the same as MK-8722, and detailed pharmacological properties including the binding pattern to AMPK and its ex vivo and in vivo effects on glucose metabolism has been recently described in detail [15]. In the current study, use of four structurally different compounds addresses whether the resultant findings were mechanism-based as opposed to compound specific. When dose-matched for similar pharmacodynamic (PD) action at C_max_, by ascertaining the change in the area under the curve (AUC) for blood glucose following an ipGTT at 1h post dose for LA1, LA2, SA1, and SA2 (all dosed at 30 mg/kg orally), these compounds were similarly efficacious (-34%, -32%, -38%, and -40%, respectively; compared to vehicle). However, the ratio of drug exposure at C_max_ relative to the trough concentration (C_trough_, i.e. plasma exposure preceding the next dose) was markedly different for the long- versus the short-acting compounds. The C_max_/C_trough_ ratios were 7.5 and 8.0 for LA1 and LA2, but were 324 and >648 for SA1 and SA2, indicative of marked dissipation of drug exposure and drug action with short-acting relative to long-acting compounds. Even with sharp differences in C_max_/C_trough_ PK the effects on blood glucose at C_trough_ were more pronounced following chronic treatment with the short-acting compounds compared to treatment with the long-acting compounds. Additional PK parameters are presented in Fig 1. In all studies, elevation of phosphorylated acetyl-CoA carboxylase (pACC) was used to as a biomarker of AMPK target engagement within skeletal muscle.

### Short-acting versus long-acting pharmacologic AMPK activation

**Fig 2:**
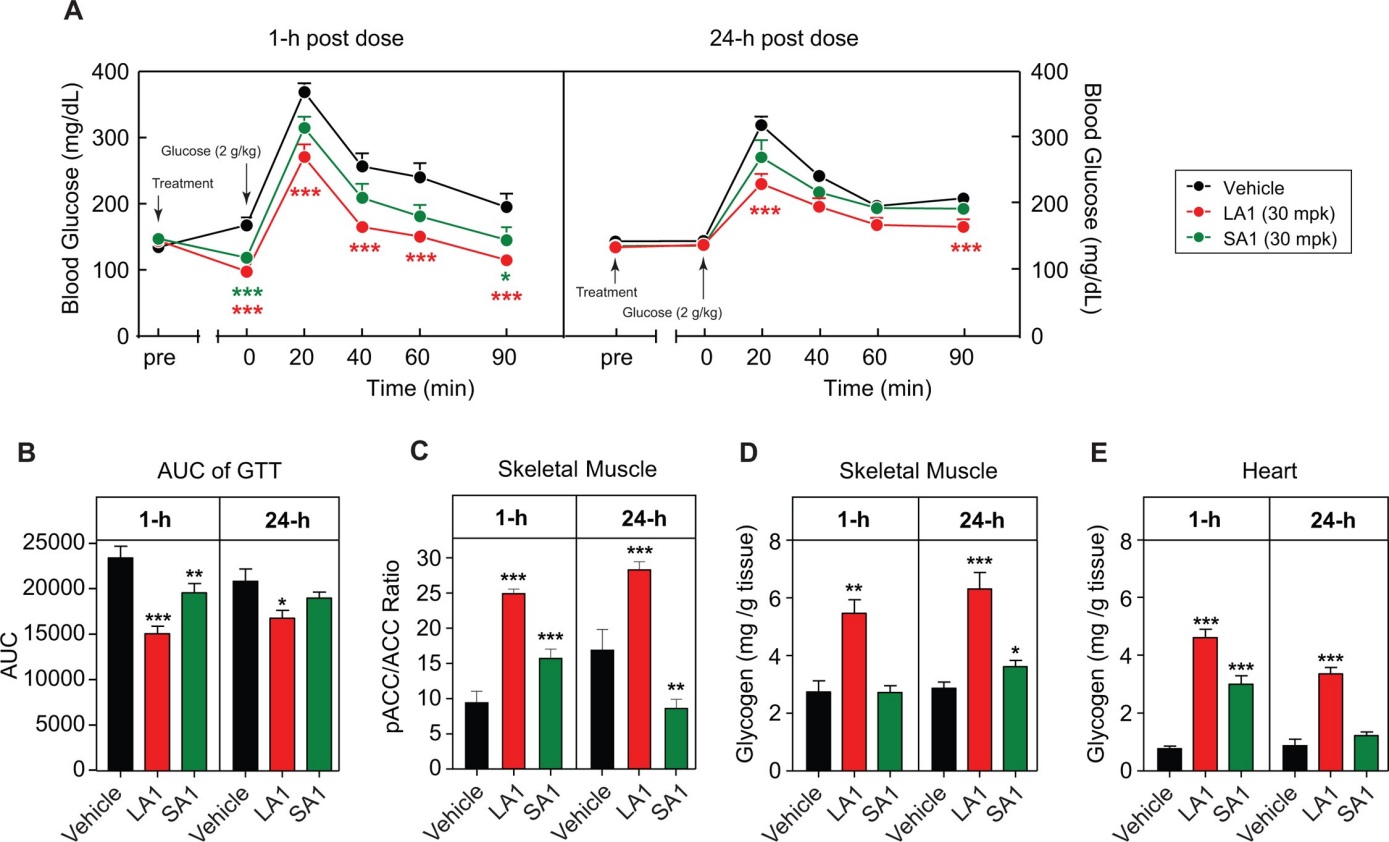
Effects of AMPK activators on glucose clearance and glycogen mobilization in lean C57BL/6 mice. Effects of LA1 (30 mg/kg, PO) and SA1 (30 mg/kg, PO) on glucose tolerance, target engagement and glycogen at 1h and 24h post-dose (n = 8). (A) Blood glucose/time curve in an ipGTT. (B) AUC of the blood glucose/time curve. (C) pACC/ACC ratio in skeletal muscle. (D–E) Glycogen contents of skeletal muscle and heart. Data are represented as mean ± SEM. *p < 0.05, **p < 0. 01, *** p < 0.001 relative to vehicle.

The first set of studies was performed to compare the effects of a long-acting AMPK activator (LA1) against a short-acting AMPK activator (SA1) to better understand the relationship between drug exposure and corresponding effects on blood glucose concentrations. Oral administration of 10 mg/kg LA1 to 8-week old db/db mice resulted in a relatively flat PK profile with sustained compound exposure up to 24h post-dose (Fig 1). A single dose of LA1 in lean C57BL/6 resulted in a reduction in fasting blood glucose, evident as early as 1h post-dose, and a reduction in glucose excursion following a glucose challenge (ipGTT) (Fig 2A). At 24h post-dose, while effects on fasting blood glucose had largely waned, circulating levels of LA1 at C_trough_ were still sufficient to suppress glucose excursion during an ipGTT (Fig 2A and 2B). Administration of the short-acting analog SA1 resulted in a similar lowering of blood glucose to LA1 at 1h post-dose. However, there was an absence of efficacy 24h post-dose (Fig 2A–2C), which correlated with the minimal C_trough_ drug exposure previously shown in Fig 1. Levels of muscle pACC in response to LA1 and SA1 mirrored these PK differences as LA1 induced significant increases at 1h and 24h post-dose whereas SA1 induced elevation of pACC at 1h but not at 24h (Fig 2C) and as will be shown below, skeletal muscle pACC was no longer increased even at 5 or 7h after SA1 administration.

The effect of LA1 to stimulate glucose uptake, glucose-6-phosphate formation and glycogen synthesis in myotubes and in vivo in rodent and rhesus monkey skeletal muscle has previously been described [15]. Short-acting and long-acting compounds have highly similar acute effect on glucose uptake into muscle but due to the sharp differences in duration of action, important differences are observed between a long-acting versus short-acting AMPK activator on tissue glycogen. LA1 increased glycogen content in skeletal muscle at 1h and 24h post dose and LA1 caused a large increase of heart glycogen, consistent with the earlier report [15]. In contrast, SA1 showed marginal induction of glycogen accretion in skeletal muscle at both time points (Fig 2D), and though SA1 increased heart glycogen at 1h, the effect was no longer evident at 24 hours, in contrast to the effects of LA1 denoting mobilization of glycogen and a return to baseline levels as drug exposure with SA1 dissipated (Fig 2E).

### Transcriptional effects of pharmacological AMPK activation and of exercise

**Fig 3:**
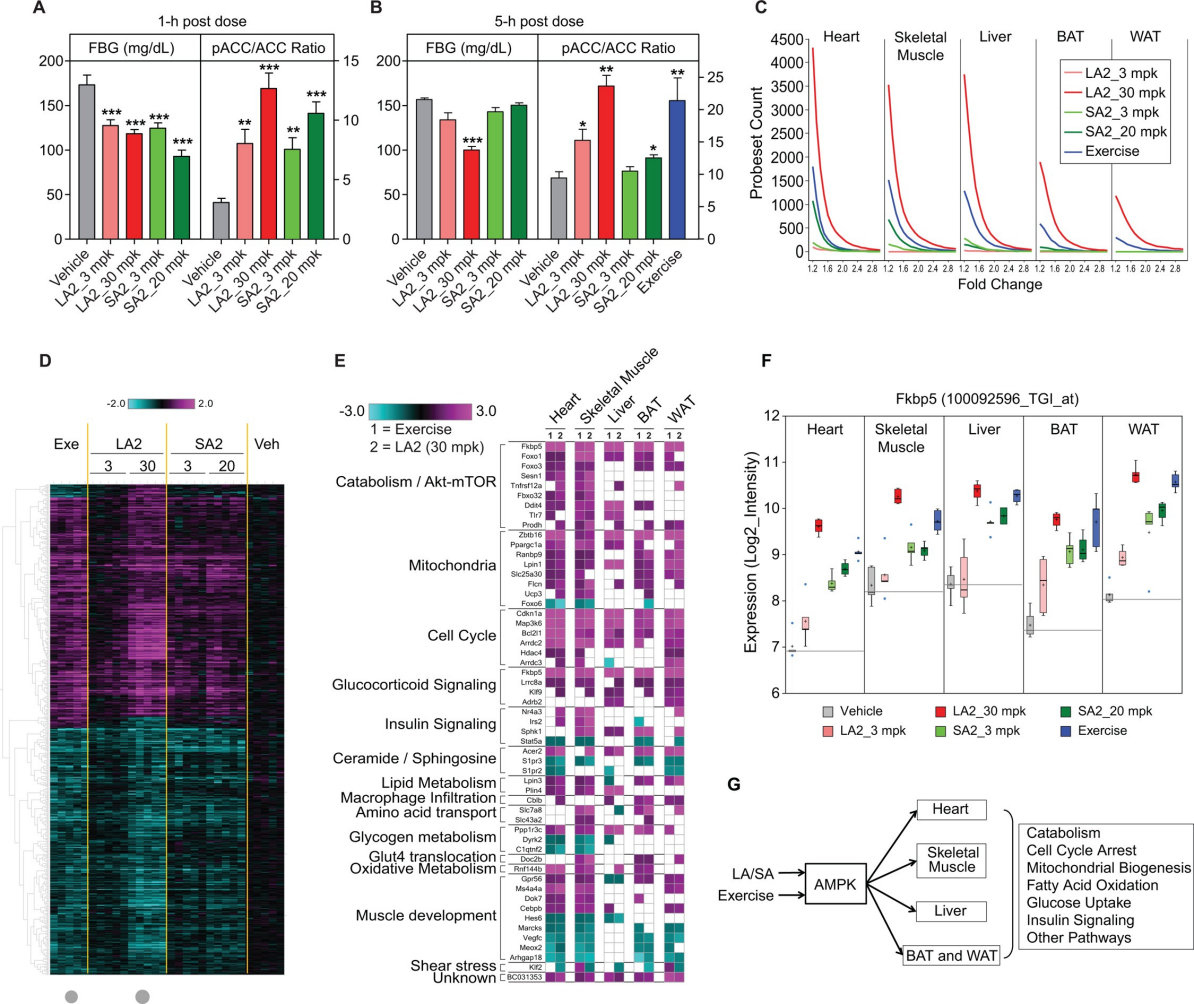
Comparison of pharmacological AMPK activation and exercise in lean C57BL/6 mice (n = 8). Effects of pharmacological AMPK activation on fasting blood glucose (FBG) and pACC/ACC ratio 1h (A) and 5h (B) post dose (LA2, 3 and 30 mg/kg, PO and SA2, 3 and 20 mg/kg, PO) in 12-week old lean C57BL/6 mice. The effects of 130 min treadmill exercise on pACC/ACC ratio (treadmill exercise at speed of 10 m/min) are also shown in (B); Data are represented as mean ± SEM. *p < 0.05, **p < 0. 01, ***p < 0.001 relative to vehicle. (C) Acute exercise and pharmacological AMPK activation have robust transcriptional effects in heart, skeletal muscle, and liver (n = 5). Tissues were collected 5 h post dose, or at the end of exercise. Shown are the number of probesets (y-axis) that met the fold change cutoffs (x-axis). Only the probesets with FDR_BH (False Discovery Rate Benjamini & Hochberg) p<0.1 were included. (D) For skeletal muscle gene expression profiling, 789 probesets are shown that met the +/- 1.2 fold change and FDR_BH p<0.1 threshold in both the exercise and LA2 (30 mg/kg, PO) groups (indicated by grey dots). The color gradient represents fold change compared to vehicle treated sedentary mice (-2.0 to 2.0 fold). (E) Pathway analysis of heart, skeletal muscle, liver, BAT, and WAT of C57BL/6 mice (1) exercised on treadmill for 130 min; and (2) treated with LA2 (30 mg/kg, PO). White boxes represent changes that did not reach statistical significance (1.2 fold and p<0.05). (F) Fkbp5 was one of six genes (represented by 7 probesets) that were significantly regulated by both acute exercise and acute pharmacological AMPK activation (LA2, 30 mg/kg, PO) in all 5 tissues profiled. Shown in the box plot are the log2_Intensity values per treatment group. (G) Schematic diagram summarizing the pathways regulated by exercise and direct AMPK activation.

A systematic examination of the transcriptional effects across skeletal and cardiac muscle, liver, white and brown adipose was conducted following the administration of a single dose of a long-acting (30 or 3 mg/kg LA2), and a short-acting (20 or 3 mg/kg SA2) activator in comparison to the effects of an acute bout of strenuous exercise (1300 m of treadmill running as described in Materials and methods). A vehicle treated, sedentary control group was included. Consistent with the PK characterization described above, the LA2 and SA2 compounds induced very similar acute effects (at 1h, Fig 3A) on blood glucose that were dose-dependent. With respect to the target engagement biomarker pACC, SA2 showed no effects after 5h, while LA2 maintained reduced blood glucose and elevated muscle pACC beyond 5h post-dose. pACC was also elevated in the skeletal muscle of mice subjected to the single bout of exercise (Fig 3B). Total RNA from heart, skeletal muscle (vastus lateralis), liver, brown and white adipose tissues was isolated from all treatment groups approximately 5h post dose and analyzed with custom Affymetrix microarrays (see Materials and methods).

The session of exercise induced a robust transcriptional response in each of these five tissues as did administration of the pharmacologic AMPK activators and for the latter, the transcriptional response was more robust with the higher dose for both SA2 and LA2 (Fig 3C). This RNA profiling showed a striking similarity between pharmacological AMPK activation and acute exercise that was evident within each of the five tissues (S4–S23 Tables and S3–S21 Figs). There was also close similarity in the response to SA2 and LA2 in each organ, as would be expected with a mechanism based rather than compound specific effect (Figs 3D and 3E and S7–S18). The sedentary animals treated with vehicle served as baseline for both the exercised group and the AMPK activator treated groups and 1-way ANOVA analysis (2-group) was performed within each tissue to generate fold change and significance statistics (raw p-values and FDR_BH corrected p values). The tissue with the strongest response in terms of the number of probesets with FDR_BH <0.1 following either exercise or AMPK activator treatment was heart, followed by vastus lateralis, liver, BAT, then WAT (Fig 3C). Gene expression signature lists were identified by applying a cutoff of 1.2-fold change and FDR_BH <0.1 and these are summarized in S4 Table. The number of probesets in the exercise and AMPK activator treatment signatures ranged from 2 to 4312 for SA2 (20 mg/kg) in WAT and for LA2 (30 mg/kg) in heart, respectively (S4 Table).

Given the acute nature of the study, the transcriptional similarities between the effects of LA2 and SA2 are not surprising. In the interests of brevity, we restrict the following discussion to compare the effects of LA2 (30 mg/kg) to those evoked by a single bout of exercise. Overall approximately half of the exercise signature within each tissue was also significantly regulated by direct AMPK activation with practically all the transcripts regulated in the same direction as reflected in the heat map in Fig 3D (with the corresponding scatter plot in S3 Fig) for the intersection signature for vastus lateralis. Additional exercise-regulated genes showed a trend toward similar regulation following direct AMPK activation however (data not shown). As described further below, there were also probesets that were specifically regulated by only one treatment, either exercise or direct AMPK activation (S4–S21 Figs and S4–S23 Tables), which could represent respectively, AMPK-independent effects of exercise and exercise-independent effects of pharmacological AMPK activation.

A considerable proportion of specific transcriptome responses regulated by exercise had the characteristic of being organ-specific rather than universal across all five tissues. Similarly, a considerable proportion of transcriptomic responses regulated by pharmacological AMPK activation were organ specific. In fact, across all 5 tissues we detected only seven probesets, representing six genes (*Fkbp5*, *Zbtb16*, *Cdkn1a*, *Map3k6*, *Bcl2l1*, and *BC031353*) that were common across all tissues, coordinately and significantly regulated by both acute exercise and acute pharmacological AMPK activation (Figs 3E and 3F and S22–S27). Nonetheless, the transcriptomic response to both exercise and pharmacological AMPK activation though revealing organ-specific differences do point to a coordinated induction of catabolism and fuel mobilization, mitochondrial metabolism, cell cycle arrest, and several additional pathways in a manner consistent with prior literature concerning the general effects of AMPK [17–23]. Interestingly, similar transcriptional effects on genes involved in regulating Akt, mTOR, Foxo, autophagy, mitophagy were observed by both AMPK activation and exercise in one or more tissues. A transcriptomic response related to muscle development was more limited in expression to heart and skeletal muscle (Fig 3E).

Importantly, the overlap of significantly regulated genes between acute exercise and acute AMPK activator treatment at the transcriptional level was larger than the robust and “unique” responses by each treatment as demonstrated by 789 probesets significantly altered by both acute exercise and acute LA2 treatment in vastus lateralis (at least 1.2-fold change and FDR_BH <0.1) (Fig 3D and S5 Table). This compares to the 62 probesets were robustly regulated only by acute exercise in this tissue (at least 1.5-fold change and FDR_BH <0.1) (S4 Fig and S6 Table). Genes involved in muscle contraction and signaling pathways were in the latter category (e.g. *Cyr61* which plays a role in Akt and integrin-mediated signaling [24], *Edn1* which regulates p38Mapk cascade [25], and *Ano1* which regulates Erk cascade and muscle contraction [26]). Conversely, 57 probesets were significantly regulated after acute AMPK activator treatment but not by acute exercise (S5 Fig and S7 Table), and no specific pathway was enriched in this set. Nevertheless, genes involved in cellular transport pathways such as Rab20 [27] and glucose-uptake in muscle such as Pikfyve [28] were specifically regulated by the AMPK activators.

### Effects of AMPK activators and exercise on glycogen accumulation and mobilization

**Fig 4:**
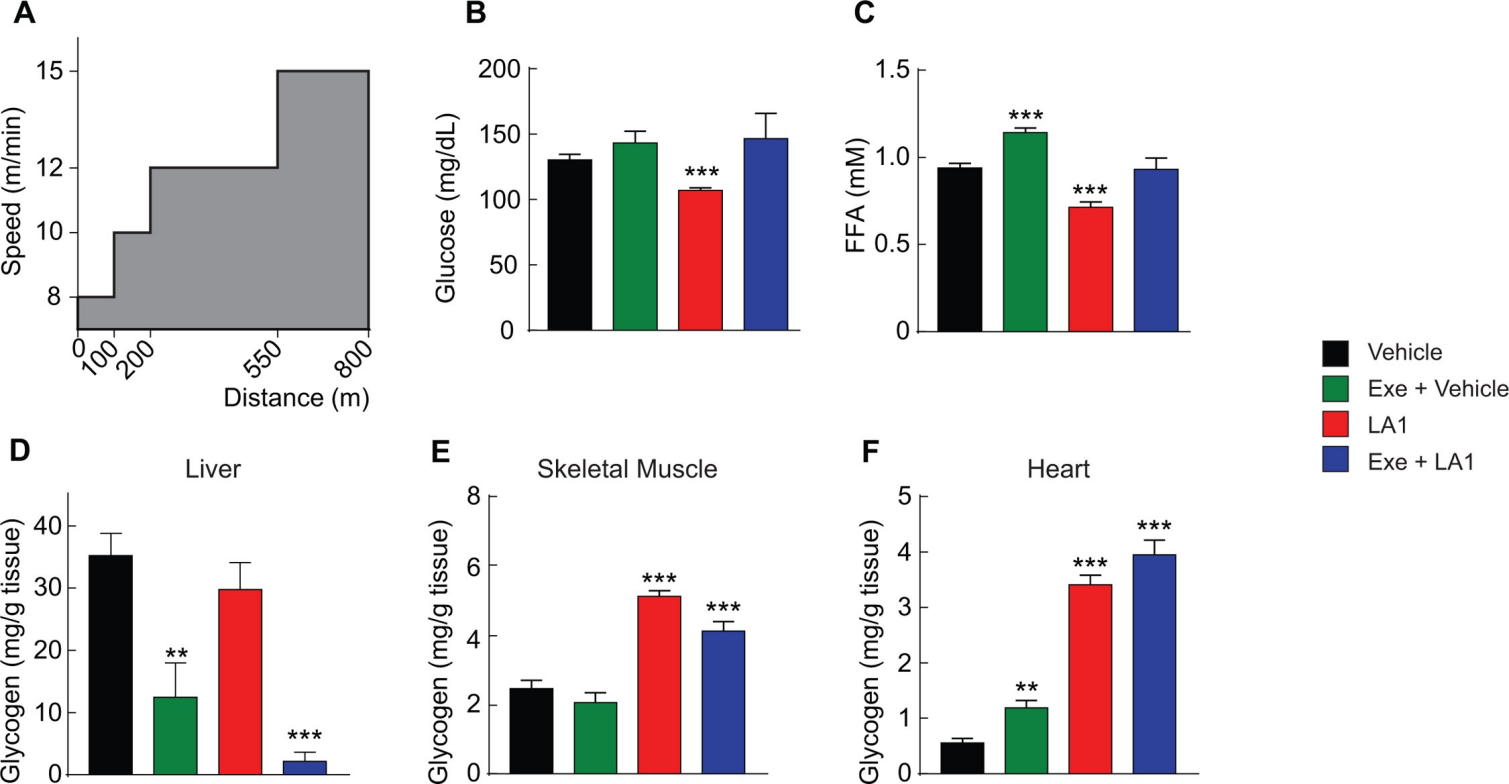
Effects of AMPK activators and exercise on glycogen accumulation and mobilization in liver, skeletal muscle, and heart. Lean mice, sedentary or exercised on treadmill, were treated with vehicle or LA1 (10 mg/kg, PO). (A) Exercise paradigm (n = 8). (B) Effects on plasma glucose. (C) Effects on plasma FFA. (D–F) Effects on glycogen contents of liver, skeletal muscle, and heart. *p < 0.05, **p < 0. 01, *** p < 0.001 relative to vehicle.

Even a single session of exercise, especially of moderate to high intensity, can improve insulin sensitivity and glucose homeostasis for a short period of time. This improvement has been attributed to energy expenditure and substrate utilization during exercise and especially the depletion of tissue glycogen during exercise [29]. To further compare pharmacological activation of AMPK with exercise, studies were done to evaluate respective effects on glucose homeostasis and tissue glycogen. In lean healthy mice, pharmacologic activation of AMPK (a single dose administration of LA1, under sedentary conditions) was compared to the effect of a single session of exercise. In addition, the combined effect of exercise and LA1 was appraised and a sedentary control group was studied. Because long- and short-acting AMPK activators do have similar acute effects (at respective C_max_) to stimulate glucose uptake and fat oxidation, a similar pattern of results might have been observed if a short-acting AMPK activator had been administered immediately following exercise, but with an important caveat that respective effects on tissue glycogen may well differ, as earlier shown during resting conditions. The single bout of exercise was 800 meters on treadmill at an increasing speed, as shown in Fig 4A. Exercise did not alter blood glucose (compared to the sedentary control group) but did significantly raise plasma FFA. In contrast, a single dose administration of LA1 slightly, but significantly reduced blood glucose and more clearly reduced plasma FFA (Fig 4B and 4C). The combination of exercise and LA1 had no net effect to alter blood glucose or plasma FFA compared to sedentary controls.

With respect to hepatic and cardiac glycogen, the single bout of exercise decreased glycogen in the liver but increased cardiac glycogen, albeit modestly (Fig 4D–4F). There was a trend towards a decrease in skeletal muscle glycogen with acute exercise. Unlike acute exercise, administration of LA1 did not induce a decrease in hepatic glycogen and it substantially increased skeletal muscle glycogen, by approximately two-fold, and increased cardiac glycogen by approximately 5-fold relative to levels in the sedentary control (Fig 4F). Combining acute exercise and administration of LA1 revealed an effect to even more completely deplete hepatic glycogen, while accretion of skeletal muscle and cardiac glycogen was like the effect of LA1 alone (Fig 4D–4F). Taken together, the findings indicate that the acute metabolic effects of pharmacological activation of AMPK does not replicate the acute metabolic responses of substrate utilization that occur during exercise, and most importantly, as will be shown below, does not recapitulate the rise in energy expenditure that occurs during exercise.

Having observed clear differences in the acute pattern of substrate utilization occurring during exercise versus that induced by pharmacologic AMPK activation, and with notable differences in the handling of glycogen and fatty acids, we further analyzed the respective transcriptional responses in the pathways of glycogen, glucose and fatty acid metabolism (S24 Table and S28 Fig). As compared to the transcriptional response to exercise, LA2 evoked a significantly greater expression in cardiac muscle for glycogen synthase 1 (Gys1) (with non-significant trends in skeletal muscle and liver) and a significantly greater expression in cardiac and skeletal muscle and liver for UDP-glucose pyrophosphorylase 2 (Ugp2) (with a non-significant trend in heart). These enzymes catalyze important steps in glycogen formation. Also, LA2 evoked greater expression of hexokinase 2 (Hk2), glucokinase (Gck), and phosphofructokinase (Pfkp) across these tissues, and the differences compared to exercise was significant in skeletal muscle and liver. Relative to the effect of exercise, LA2 evoked a significantly higher expression in cardiac muscle for hydroxyacyl-CoA dehydrogenase (Hadha) and in skeletal muscle, for acyl-CoA synthetase long chain family member 6 (Acsl6), which could be associated with higher rates of fatty acid utilization and hence, lower plasma fatty acids in response to pharmacologic AMPK activation versus exercise and act to spare utilization of muscle glycogen. More research is needed to interrogate protein translation and functional impact of these transcriptional differences, but the findings support the hypothesis that the responses to pharmacological AMPK activation disposes to glycogen formation which is consistent with the observed differences in patterns of glycogen utilization between exercise and long-acting pharmacological AMPK activation.

### Effects on glucose homeostasis by daily administration of short-acting AMPK activators

**Fig 5:**
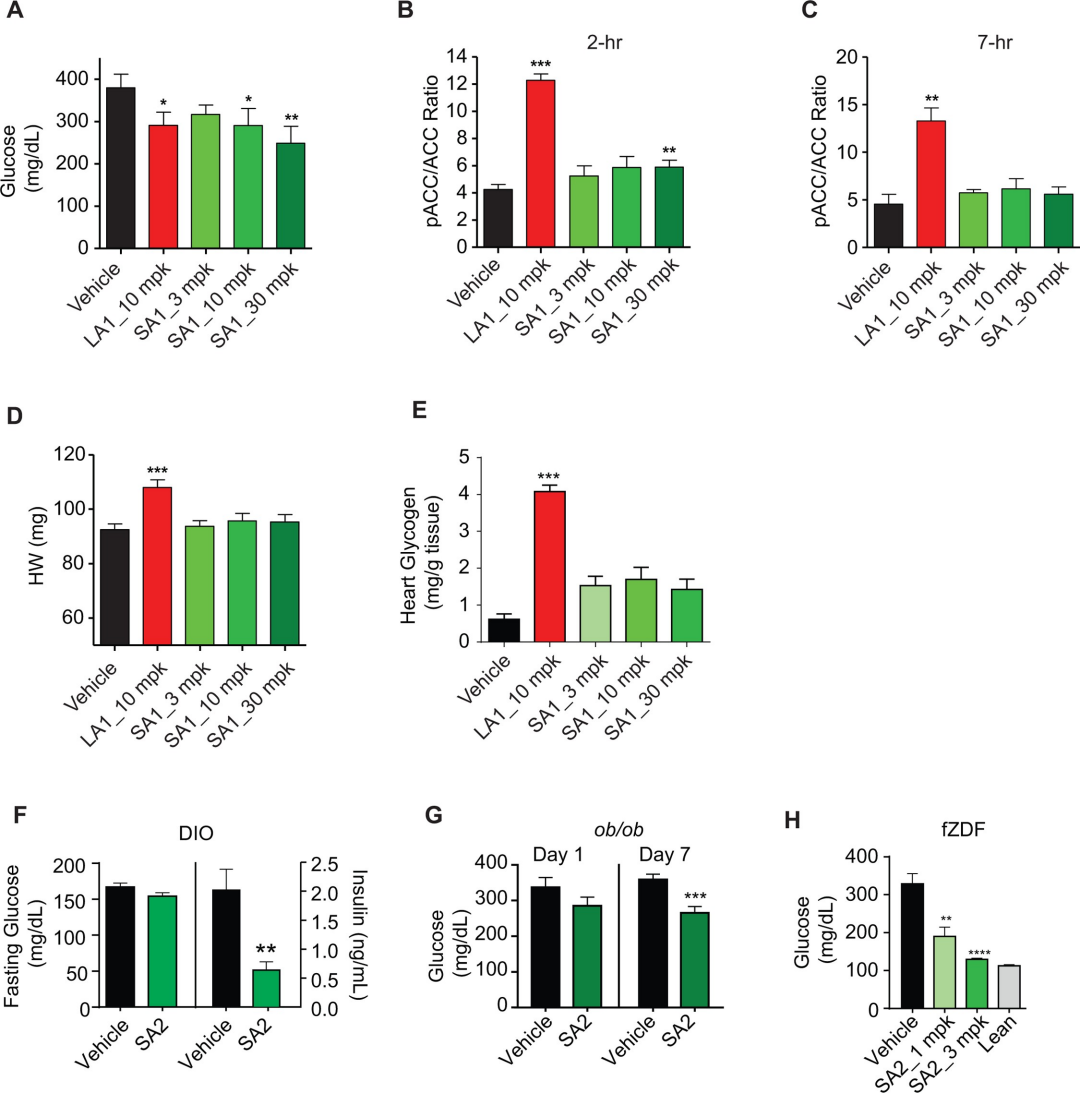
Short-acting AMPK activators reduce hyperglycemia, at trough, without inducing cardiac hypertrophy in rodent models of diabetes; db/db (A-E), eDIO (F), and ob/ob (G) mice and fZDF (H) rats. (A) Glucose measured at trough (approximately 24 hours post last dose) on day 12 of 8 week old male db/db mice treated with vehicle, LA1, and SA1 (n = 10). (B–C) pACC/ACC ratio of skeletal muscle at 2h (n = 7) and 7h (n = 3) post dose on day 14. (D) Heart weight of db/db mice on day 14. (E) Heart glycogen content of db/db mice on day 14. (F). Plasma levels of fasting glucose and insulin in 26 week old eDIO mice (average body weight of 48 grams) treated with vehicle or SA2 (3 mg/kg, QD, PO) for 4 weeks (n = 7). (G) Glucose levels of 7 week old ob/ob mice at 24h post compound administration on day 1 and day 7. (H) Glucose levels at 24h post dose (trough) after 21-day treatment of SA2 (1 and 3 mpk, QD, PO) in 8 week old fZDF (n = 10) and lean control rats (n = 6). Data are represented as mean ± SEM. *p < 0.05, **p < 0. 01, *** p < 0.001 relative to vehicle.

Having observed that a short-acting AMPK activator achieves only transient target engagement and short-lived stimulation of glucose transport yet does evoke a transcriptional response across tissues strongly congruent to exercise, we next explored whether repeated daily administration of a short-lived AMPK activator might yield disease-modifying effect on diabetes and insulin resistance. As earlier reported [15, 16], long-acting AMPK activators do exert disease-modifying effect on diabetes, yet mechanistically this has been attributed to sustained stimulation of glucose transport into skeletal muscle. Thus, the set of studies with the short-acting AMPK activators tests the hypothesis that induced transcriptional effects are sufficient to exert salutary disease modification in diabetes and insulin resistance. Two-week treatment of db/db mice, a model of T2DM with severe insulin resistance, was done with a long-acting compound (LA1, 10 mg/kg, QD) or a short-acting compound (SA1, 10–30 mg/kg, QD). Both LA1 and SA1 elicited significant reductions in non-fasting blood glucose (Fig 5A), an effect evident with repeated daily SA1 administration even at time points when circulating concentrations of drug were very low and tissue pACC was no longer affected (Fig 5B and 5C). With the long-acting compound, the improvements in hyperglycemia were accompanied by significant increases in heart weight and cardiac glycogen content, as previously noted [15]. However, chronic administration of the short acting compound did not significantly cause cardiac hypertrophy and did not increase cardiac glycogen content (Fig 5D and 5E).

To further investigate the potential disease modifying effects of chronic administration of a short-acting AMPK activator, three additional insulin resistant models were examined; including diet induced obese (DIO) mice, ob/ob mice and female Zucker diabetic fatty (fZDF) rats. DIO mice do not manifest fasting hyperglycemia but have elevated insulin, and after treatment with SA2 (3 mg/kg) for 4 weeks, when measured 18h post dose, there was a significant reduction in plasma insulin and a trend towards decreased fasting plasma glucose (Fig 5F). Daily administration of SA2 for only 7 days to ob/ob mice resulted in a progressive decrease in non-fasting blood glucose levels (Fig 5G). In this study, blood glucose was measured at 24h post dose, when drug level was extremely low. In a study conducted in fZDF rats with two cohorts, receiving respectively relatively low doses of a short-acting AMPK activator administered daily for 28-days (SA2 at 1 and 3 mg/kg, QD), a dose-dependent decrease in blood glucose was observed when measured at a time of trough drug exposure (Fig 5H). Body weight was not affected by compound administration. Taken together, these four studies, that ranged from 7 to 28-days of daily administration of a short-acting AMPK activator, despite only short-duration daily stimulation of glucose transport, nonetheless exerted disease-modifying efficacy. The effect with chronic administration of a short-acting AMPK activator to improve hyperglycemia and hyperinsulinemia implicate mechanisms other than a sustained pharmacologic stimulation of glucose transport into skeletal muscle.

### Effects of AMPK activation on fatty acid oxidation (FAO) and energy expenditure

**Fig 6:**
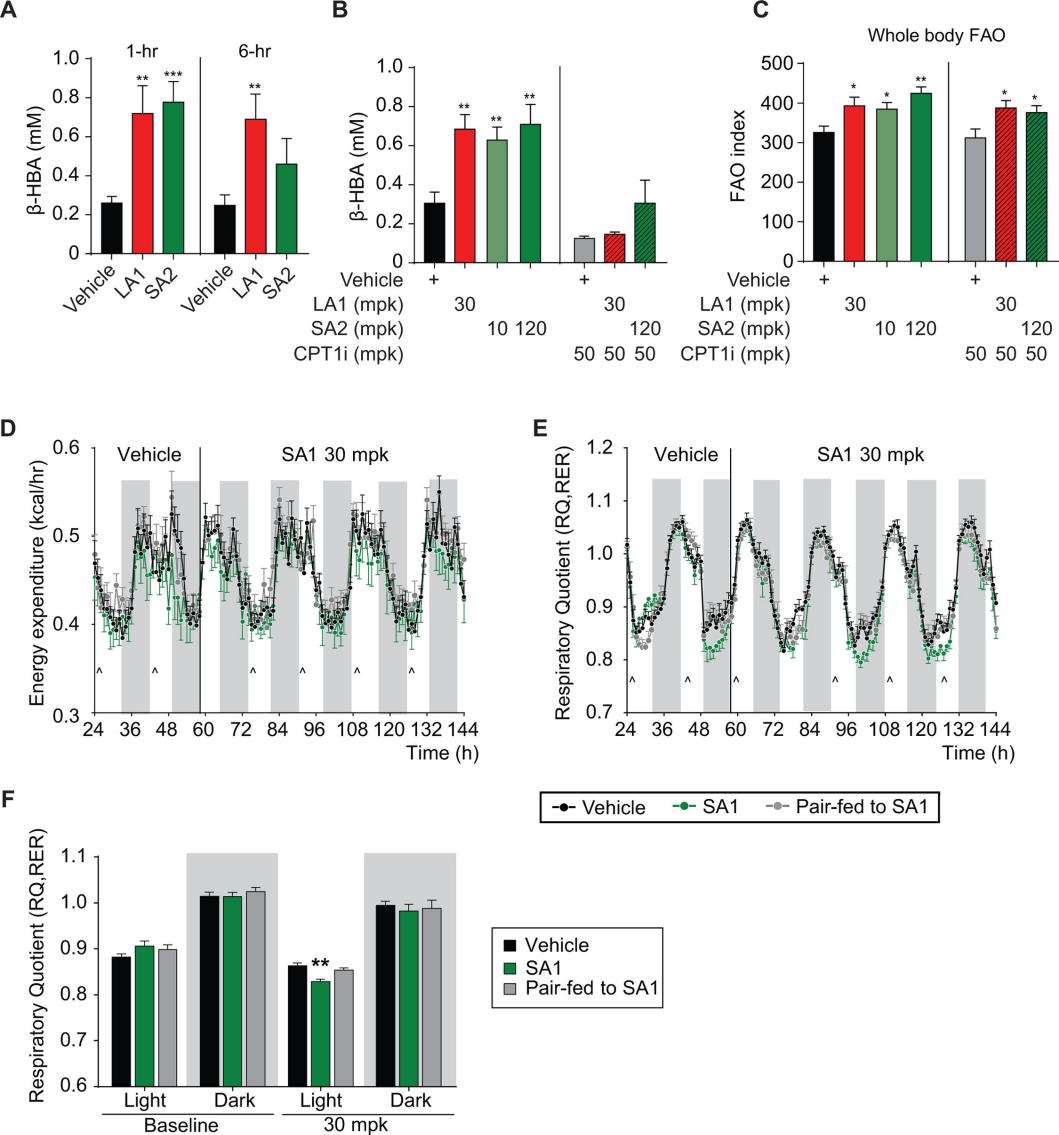
Effect of AMPK activators on FAO in db/db mice (A-C) and energy expenditure, respiratory exchange ratio and FAO in lean C57BL/6 mice (D-F, n = 8). (A–C) Plasma level of β-hydroxybutyrate and FAO index determined by D2O-labeled oleate oxidation in 8 week old db/db mice treated with vehicle, LA1 (30 mg/kg), SA2 (30 mg/kg (A), 10–120 mg/kg (B-C)), CPT-1 inhibitor (CPT-1i) (50 mg/kg), and SA2 (120 mg/kg) + CPT-1i (50 mg/kg), all QD, PO. (D) Time curve of whole body energy expenditure in 12 week old lean C57BL/6 mice (average body weight of 25 grams) housed in metabolic cages at baseline and after treatment with vehicle (0.25% MC, 5% Tween-80, 0.02% SDS, 5mM HCl, and 60mg/kg HPMCP polymer) or SA1 at 30 mg/kg (QD, PO indicated by arrows) or mice pair-fed to the SA1 group. (E—F) Respiratory quotient (RQ, or respiratory exchange ratio, RER) in mice after treatment with vehicle or SA1 at 30 mg/kg (QD, PO indicated by arrows) or mice pair-fed to the SA1 group. Data are represented as mean ± SEM. *p < 0.05, **p < 0. 01, *** p < 0.001 relative to vehicle.

Exercise training enhances muscle oxidative capacity and fatty acid oxidation [30]; the latter attributed acutely to pAMPK-mediated phosphorylation and inhibition of ACC with subsequent reduction in malonyl-CoA [31], as well as the transcriptional response enhancing mitochondrial content and function. Across several rodent and murine models, we examined the impact of pharmacologic AMPK activators on fatty acid oxidation (FAO). Treatment with LA1 or SA2 resulted in a significant acute increase in hepatic FAO reflected by circulating β-hydroxybutyrate (βHBA) levels at one hour; however, this effect was no longer significant at 6 hours with SA2 but did persist with LA1 (Fig 6A). Consistent with the expected hepatic source for βHBA, the effect of LA1 or SA2 on plasma βHBA was completely ablated by concomitant treatment with a liver-restricted/targeted carnitine palmitoyltransferase I (CPT-1) inhibitor (CPT1i) (Fig 6B). Administration of LA1 for 21 days to eDIO mice reduced liver triglyceride content by 56% (p<0.001 vs vehicle, S30A Fig) without decreased body weight, which was similar to the effect of SA2 (40% reduction in liver triglyceride content—S30B Fig). Associated with this was significant reductions in plasma TG and apoB concentrations (S30C and S30D Fig). Thus, sustained target engagement by an AMPK activator in the liver is not required in order to achieve improvement in hepatic steatosis and dyslipidemia.

The impact of LA1 or SA2 on whole body FAO was estimated by measuring D_2_O production from systemically administered deuterated free fatty acid (FFA). Acute treatment with LA1 or SA2 led to a significant increase in D_2_O production signifying increased FAO. Co-dosing of LA1 or SA2 with the liver-targeted CPT1 inhibitor revealed only a modest decrease in generation of D_2_O, indicating a sizeable proportion of systemic FAO stimulated by LA1 or SA2 was occurring in extra-hepatic tissues, likely including muscle (Fig 6C). Elevated plasma and tissue acylcarnitine concentrations, a biomarker of incomplete FAO have been associated with mitochondrial dysfunction and insulin resistance [32, 33], and levels change in response to exercise [34]. With chronic daily dosing of SA2, there were significant decreases in concentrations of plasma acylcarnitines evident across numerous species and chain lengths, as shown in S1 Fig, indicative of an effect to induce more complete β-oxidation of fatty acids. A similar effect to reduce numerous species/chain lengths of acylcarnitines was found in cardiac tissue after chronic dosing of SA2 (S1 Fig). Again, endpoints were assessed at time points where exposures of the short acting compound were minimal (drug trough).

Chronic effects on energy expenditure and fuel utilization were assessed by treating mice housed in metabolic cages with SA1 (30 mg/kg/day) for 5 days. A similar study was also conducted using LA1 for a longer duration and with an escalating dose (3, 10 and 30 mg/kg/day). There was no change in energy expenditure during treatment with SA1 (Fig 6D) but there was a reduction in the respiratory exchange ratio (RER), denoting increased reliance upon fat oxidation, that occurred during the light cycle when animals are inactive and not eating. There was a return to control levels during the dark cycle denoting a shift toward reliance upon carbohydrate oxidation when feeding occurs. This effect to increase FAO may have represented an acute pharmacological effect of SA1, because this pattern recurred with each dose administration and without significant effects when SA1 was at trough concentrations. (Fig 6E and 6F). However, administration of LA1, despite its longer duration of exposure, had a similar effect as SA1 to lower RER mostly during the light cycle and yet not affect the shift to predominately carbohydrate oxidation with a resumption of feeding during the dark cycle (S2A Fig). In an extension of this study, continuing 30 mg/kg/day LA1 at thermoneutrality (housing temperature of 30–32°C as compared to standard sub-thermoneutral temperature of 22–26°C), a more pervasive shift to increased FAO was observed. Consistent with previous results with LA1 [15], there was no change in body weight during the study with SA1 or LA1, nor an influence of SA1 on food intake relative to control (S2B Fig).

## Discussion

AMPK is one of the crucial cellular signaling nodes activated in response to bio-energetic demand [35] and it is activated during physical activity, particularly moderate and higher intensity exercise [2]. It has further been postulated that activation of AMPK is a key cellular mechanism by which physical activity confers cardiovascular and metabolic health benefits [7]. Accordingly, pharmacological activation of AMPK warrants consideration as a potential exercise mimetic [6]. In the current studies, newly developed, potent pan-isoform activators of AMPK with excellent cell permeability characteristics that enable broad tissue distribution, as previously described [15], were used to address this question. The effect of a single bout of exercise was compared to pharmacologic AMPK activation for respective impacts on patterns of substrate utilization and upon gene transcription responses, the latter assessed across five organs. Across these studies we employed several distinct molecular entities that are potent direct AMPK activators, each able to engage both β1 and β2 containing AMPK isoforms (i.e. pan-activators), the latter critical for engagement in skeletal muscle. Use of structurally diverse activators serves to clarify whether pharmacologic effects are mechanism-based rather than idiosyncratic to a single compound and thus a potentially off-target effect. Also, by employing activators that differed in duration of action, we examined the potential for disease modification by repeated administration of a short-acting AMPK activator, a pharmacologic intervention intuitively like the short duration of an exercise session and that differed from the long-acting AMPK activators in respective effect on glycogen accumulation in heart and skeletal muscle. The short-acting compounds had only a transient effect on glucose transport and little effect on glycogen accretion whereas the long-acting compounds led to substantial glycogen accretion, an effect consistent with sustained activation of AMPK [36].

The metabolically beneficial effects of exercise can be broadly divided into short-lived benefits that derive from energy expenditure and substrate utilization during exercise [37] and to training effects, deriving from exercise-induced transcriptional expression to modify metabolic capacities [38]. Physiologically, these two lines of benefits intertwine to improve metabolism but to evaluate pharmacologic AMPK activation as an exercise mimetic we endeavored to appraise each separately. Differences in substrate utilization during exercise versus pharmacologic AMPK activation were observed. Treadmill-based running by lean mice activated muscle AMPK and reduced liver glycogen. In contrast, acute pharmacologic AMPK activation (under sedentary conditions), using a long-acting compound, acted to increase muscle glycogen more than 2-fold, in heart by more than 3-fold, and with less mobilization of hepatic glycogen than exercise. Though pharmacologic AMPK activation does appear to mimic many of the cellular actions signaled by exercise-induced activation (e.g. stimulation of glucose transport and fatty acid oxidation), the net effect especially on glycogen balance is quite different from exercise and for a fundamental reason. Exercise increases energy expenditure and substrate oxidation in an intensity and duration dependent manner [39]; for example, moderate-intensity exercise achieves a 3 to 6-fold increase of METs (multiples of resting energy expenditure). In contrast, a direct acting pharmacologic AMPK activator, as demonstrated in the current studies, regardless of whether long-acting or short-acting in duration, does not change resting energy expenditure. This fundamental difference in energy balance between exercise versus pharmacologic AMPK activation shaped the respective differences in substrate balance and from this perspective, pharmacologic AMPK activation should not be viewed as an exercise mimetic. Arguably, for a patient population who would warrant treatment with an exercise mimetic due to inability to undertake physical activity, a pharmacologic approach that acutely increased energy expenditure by 3 to 6 METS might entail untoward cardiovascular effects.

When comparing respective effects on gene transcription however, there was a striking concordance between exercise and pharmacologic AMPK activation. The high degree of similarity in gene expression induced acutely by exercise and by pharmacologic AMPK activation was robustly evident across five tissues: skeletal muscle, heart, liver, white adipose and brown adipose. To our knowledge, the profiling of the transcriptomic response to exercise across these five tissues simultaneously is itself a novel and valuable set of data, comprising a more complete cross-organ portrayal of gene transcription induced by exercise than has previously been reported. With regard to the pharmacological activators, as earlier noted, these are “pan-activators” of all twelve APMK isoforms and demonstrate good bio-distribution and cell permeability characteristics [15], so an assumption of this aspect of the study is that the transcriptomic responses to the pharmacological AMPK activators represented a direct drug effect across all of these five tissues. A caveat, however, that should be acknowledged is that the degree of AMPK activation though measured in liver, skeletal muscle and heart for LA compounds, and in liver and skeletal muscle for SA compounds, was not measured in adipose tissues, either in response to exercise or to the administration of the pharmacological AMPK activators. As expected, there was considerable organ-specificity to the gene expression changes induced by either exercise or direct AMPK activation. Even so, within each tissue, the majority of the exercise signature was very similarly regulated by pharmacologic AMPK activation. Within the shared transcriptomic response were pathways regulating glucose and lipid metabolism, mitochondrial biogenesis, cell cycle regulation, and numerous other pathways, as shown in Fig 3G. These are pathways well recognized to be governed by the activation of AMPK [40]. Also consistent with a mechanism-based pharmacological effect, transcriptomic responses were highly similar for a long-acting and a short-acting activator and were dose-responsive. AMPK is known to have a crucial role in governing metabolism not only in skeletal muscle, but also in the heart, in adipose tissues and in the liver [41–43]. The gene transcription data obtained in the current studies support the concept that physiological AMPK activation is a crucial node governing the response to exercise, not just in skeletal muscle, but also in liver, adipose (brown and white) and heart.

We also carefully examined the differences in transcriptomic response to exercise versus pharmacologic AMPK activation. In skeletal muscle, of the 62 probe sets regulated only by acute exercise, the genes were mostly involved in muscle contraction, a physiological process not elicited by pharmacologic AMPK activation. Of the 57 probe sets that were significantly regulated only by the AMPK activator, no specific pathway was enriched but individual genes such as Pikfyve, which is a known AMPK target and is involved in glucose uptake, were regulated. A further targeted comparative analysis between responses to exercise and pharmacologic AMPK activation was undertaken in the context of observed differences in glycogen metabolism. Pharmacologic activation of AMPK evoked stronger gene expression than did exercise for glycogen synthase 1, UDP-glucose pyrophosphorylase 2, hexokinase 2, and glucokinase, each an important step in the pathway for glucose uptake and glycogen formation. Thus, even with the respective differences noted, from a perspective of comparative gene transcription effect, pharmacologic AMPK activation largely recapitulates the effect of exercise.

There has been a long-standing interest in the potential of AMPK as a drug target for the treatment of type 2 diabetes mellitus and related metabolic syndromes [44–46]. Two recent publications on pharmacological AMPK activation have more fully examined the potential as a therapy for diabetes mellitus and the mechanisms of action for improvement of hyperglycemia in animal models of diabetes mellitus and insulin resistance. Cokorinos et al [16], demonstrated that a compound selective for activation of only β1-containing isoforms of AMPK (thus not engaging isoforms in muscle) does not meaningfully improve hyperglycemia, whereas a compound capable of activating both β1- and β2-containing isoforms of AMPK does. The β1/β2 activator stimulates glucose transport in skeletal muscle. In the other recent study on pharmacological AMPK activation, from our group, Myers et al [15], used MK-8722 (LA1 in the current studies), which is a long-acting β1/β2-AMPK activator and it significantly improved hyperglycemia. Both of these studies led to the hypothesis that sustained stimulation of glucose transport into skeletal muscle is a crucial underlying mechanism for improvements of hyperglycemia, a hypothesis that is re-examined in the current study. The study by Myers et al, included a careful examination of the effects on cardiac muscle, and found marked glycogen accretion and an induction of cardiac hypertrophy. Briefly, as reported in that manuscript [15], the increase in cardiac weight was associated with increased glycogen content within myocytes, was bi-ventricular, progressed with duration of treatment, and was dose-dependent but reversible on drug withdrawal. This pattern of response in the heart was observed to occur in mice, rodents and in non-human primates. It is noteworthy that a normal cardiac ejection fraction was maintained. The increase in heart weight though associated with an increase in cardiac glycogen cannot however be completely accounted for by the increase in glycogen. In the current study, glycogen accounted for approximately 2% of the increased heart weight with LA1 treatment. Thus, while increases of cardiac glycogen are an important biomarker of the effect in the heart of sustained pharmacologic AMPK activation, glycogen per se accounts for a relatively minor proportion of the increase in heart weight, as previously emphasized [15]. Further investigation is needed to more deeply understand the cardiac response to a long-acting AMPK activator and in particular to understand the effect on cardiac myocyte cell signaling. However, the observation of cardiac hypertrophy raises serious caution about potential adverse consequences of sustained pharmacological AMPK activation especially in the patient population with diabetes mellitus that has a high prevalence of underlying cardiac disease. Moreover, activating mutations of AMPK have been associated with glycogen accumulation in cardiac myocytes and cardiac dysfunction [47].

In the current studies, we provide novel information on the effect of short-acting β1/β2 AMPK activators, distinguished from the long-acting compounds in that these only transiently stimulated glucose uptake and minimally influenced glycogen accretion in skeletal and cardiac muscle, findings consistent with the short duration of pharmacologic target engagement. Repeated administration of the short-acting AMPK activators did not cause cardiac glycogen accumulation and importantly did not induce cardiac hypertrophy. As discussed above, the accretion of cardiac glycogen can be viewed as a valuable biomarker but per se accounts for a relatively small proportion (approximately 2%) of the overall increase of cardiac weight that has been observed with sustained pharmacological AMPK activation. An absence of accretion of cardiac glycogen with repeated short-duration pharmacological AMPK activation is potentially encouraging, but clearly, more investigation is needed to fully appraise the potential for cardiac safety of a short-acting versus a long-acting AMPK activator, including in-depth molecular analyses.

Yet, despite only transient stimulation of glucose uptake into muscle, with repeated daily administration, the short-acting AMPK activators were also shown to progressively improve hyperglycemia and hyperinsulinemia in four rodent models of diabetes and insulin resistance, measured at times of nominal or absent drug exposure. Thus, the metabolic improvements did not derive from a sustained stimulation of glucose uptake into skeletal muscle, as had earlier been postulated to be essential for the ability of long-acting AMPK activators to reduce hyperglycemia [15, 16]. As earlier described, the short-acting and long-acting AMPK activators evoked highly similar transcriptomic responses, responses strongly congruent to those evoked by exercise. Our interpretation is that at least in part, and perhaps mostly, the cumulative effect of repeated transcriptomic responses induced by short-acting pharmacologic AMPK activation underlies its disease-modifying benefits in rodent models of diabetes mellitus and obesity.

In the current study, the short-acting AMPK activators were demonstrated to acutely stimulate fatty acid oxidation in liver and non-hepatic tissues. This is a pharmacologic action consistent with known physiological actions of AMPK [14, 44]. Collateral to the improvement of hyperglycemia and hyperinsulinemia resulting from repeated daily administration of short-acting activators were numerous indices of improved fatty acid and lipid metabolism. We postulate that disease modification with repeated daily dosing of short-acting pharmacologic activation of AMPK, may in addition to the contribution from gene transcription effects also derive from this acute effect on fatty acid and lipid metabolism. Together the effect to stimulate fatty acid oxidation likely acts in confluence with the cumulative tissue re-modeling effects induced by the daily transcriptional responses in skeletal muscle, adipose tissue and liver. In relation to the two recent studies on efficacy of pharmacological AMPK activation to improve hyperglycemia [15, 16], that both emphasized as an underlying mechanism of action sustained stimulation of glucose transport into muscle, the current findings provide new perspective with regard to AMPK as a drug target. The current findings indicate that induction of salutary disease modification can be achieved by a daily administration of only short-lived activation of AMPK, and this approach may importantly avoid excess glycogen accumulation and cardiac hypertrophy that was consistently observed with sustained AMPK activation.

Lipotoxicity is one of the key pathophysiological characteristics of obesity, type 2 diabetes mellitus and insulin resistance [48], and associated with this is an impaired mitochondrial functional capacity [49]. Physical activity intervention in sedentary individuals with obesity and type 2 diabetes mellitus improves mitochondrial content and insulin sensitivity in skeletal muscle, and especially when combined with moderate weight loss substantially improves lipotoxicity [50, 51]. The current findings in rodent models indicate that short-acting pharmacologic activation of AMPK evokes metabolically favorable effects on lipotoxicity and fatty acid metabolism. More research is needed to further investigate mechanisms by which short-acting AMPK activators progressively exert beneficial disease modification, to more closely examine whether these progressive effects in skeletal muscle, liver and adipose tissues indeed resemble the training effect of physical activity on tissue re-modeling, and to explore metabolic cross-talk amongst organs versus cell autonomous contributions to disease modification. It is also conceivable that pharmacologic actions of the AMPK activators within the central nervous system contributed to the observed effects. These compounds have permeability and other chemical properties that enable broad distribution into numerous organs, including the CNS. However, no effect upon food intake was observed and AMPK activation in the CNS has been associated with increased appetite [52]. Selective, potent, direct-acting AMPK activators, with contrasting pharmacologic characteristics (e.g. isoform selectivity and duration of action), the structures of which have now been published [15, 16] and are presented in this report and can accordingly be synthesized to enable continued research.

In summary, we observed that short-acting pharmacologic AMPK activators can lead to glucose lowering efficacy and improved fatty acid catabolism when administered daily in rodent models of hyperglycemia and insulin resistance. This favorable disease-modification did not cause increased deposition of cardiac glycogen that had been found with long-acting AMPK activators. Short-acting pharmacologic AMPK activators, like long-acting ones, induce a pattern of gene expression in skeletal muscle, heart, liver and adipose tissues in rodents that is highly concordant to the effect of moderately strenuous exercise, a transcriptional resonance that occurred despite differences from exercise in respective acute substrate utilization. We postulate that a pharmacologic approach of daily short-acting AMPK activation meets several criteria that warrant its consideration as an exercise mimetic and could provide benefit, at least partially, for those individuals who are otherwise unable to engage in regular physical activity.

## Materials and methods

All animal procedures were reviewed and approved by the Institutional Animal Care and Use Committee of Merck & Co., Inc., Kenilworth, NJ USA.

### Compounds

All compounds were provided by MRL (Rahway, NJ) (structures shown in Fig 1). LA1 (MK-8722) [15] and LA2 are AMPK activators that elicit long duration of action following oral dosing while SA1 and SA2 elicit shorter duration of action. At doses used in the current studies that result in similar unbound peak plasma drug exposure, SA1 and SA2 administration results in much lower trough drug exposure vs. LA1 and LA2 (data shown in Fig 1). Synthetic procedures for all compounds can be found in the WO2012116145 (LA1 –example 159, LA2 –example 190, SA1 –example 163, SA2 –example 171).

### Animals

Male lean C57BL/6 and eDIO mice were purchased from Taconic (Hudson, NY) at 8 and 16 weeks of age, respectively. Male db/db and B6.V-Lepob/J (ob/ob) mice were purchased from Jackson Laboratory (Bar Harbor, ME) at 6 weeks of age and treatment was started at 8 weeks of age. Female Zucker Diabetic (fZDF) rats were obtained from Charles River Laboratories (Strain# 370-obese, Wilmington, MA) at 5 weeks of age. Lean C57BL/6 and db/db mice and fZDF rats were maintained on chow diet (Teklad 7012, Research Diets, Inc., New Brunswick, NJ) and eDIO mice were fed with high fat diet (D12492, 60% kcal% fat, Research Diets, Inc, New Brunswick, NJ) with free access to water. fZDF rats were switch to high-fat diet (D12451, Research Diets, Inc, New Brunswick, NJ) to induce diabetes. The plasma insulin level of db/db mice ranged from 4.8–29.6 ng/ml (11.5 ng/ml ± 2.5, n = 10), which was significantly higher than that in lean control mice (1 ± 0.3 ng/ml, n = 7). All animals were maintained in a 12h light/12h dark cycle.

Animals were acclimated to PO QD dosing with vehicle (0.25% MC, 5% Tween-80, 0.02% SDS) for approximately 1 week prior to the start of treatment with compounds and doses as indicated. Food intake and body weight were monitored during the study. Fasting glucose was determined using tail blood by glucometers (Accu-Chek, Roche). A separate drop of blood was also collected from each animal to measure fasting insulin levels (7–8 hr fasting). Intraperitoneal glucose tolerance tests (ipGTT), and determination of glycogen levels, were performed as previously described [15]. At takedown, animals were euthanized by either cervical dislocation or CO2 inhalation.

### Exercise and *in vivo* studies in mice

For the RNA profiling study, lean C57BL/6 mice were fasted by removing food at 7:30 am and then dosed with vehicle or AMPK activators by oral gavage at around 9:30 am, and kept sedentary for the remainder of the study period. For the exercise arm, mice were put on a treadmill (Columbus Instruments International, OH) for exercise with speed set at 10 meter/min. Mice exercised for 30 min followed by 30 min rest and the total running distance was 1300 meters. All mice were euthanized at around 3:00 pm and liver, heart, WAT, BAT, and skeletal muscle (vastus lateralis) were collected by freezing clamp for pACC/ACC assay (Mesoscale, MD), and in RNA later (Life Technologies, NE) for gene expression profiling. Fasting blood glucose was measured at 1h or 5h post compound treatment. pACC assay was performed by using the same procedure published previously [15].

For comparing exercise and AMPK activator treatment upon patterns of substrate metabolism, male C57BL/6 mice at 12 weeks of age were fasted by removing the food at 7:30 am. At 1:00 pm, mice were divided into 4 groups (n = 8), two groups were sedentary and two groups were exercised on treadmill at increasing speeds for a total distance of 800 meters (8 m/min for 100 m, 10 m/min for 100 m, 12 m/min for 350 m, and 15 m/min for 250 m) [53]. Animals were then treated with vehicle or LA1 (10 mg/kg, PO). Mice were euthanized at 1 hr post treatment. Plasma glucose and FFA were determined by using colorimetric kits (Wako Diagnostics and Roche, respectively). Tissue glycogen contents were determined by using the same procedure described previously [54].

### RNA isolation and gene expression profiling

RNA isolation and microarray analysis was performed as previously described [55]. Briefly, hybridization on custom mouse Affymetrix GeneChip microarrays (GEO platform GPL9734) (Santa Clara, CA.), labeling and scanning using Affymetrix ovens, fluidics stations and scanners were performed according to the protocols recommended (NuGEN, San Carlos, CA). Sample amplification, labeling, and microarray processing were performed by the Covance Genomics Laboratory in Seattle, WA. The raw gene expression data has been deposited into the Gene Expression Omnibus database (series record GSE92719; https://www.ncbi.nlm.nih.gov/geo/query/acc.cgi?acc=GSE92719). The Affymetrix chip ID numbers are listed in S2 Table with the number of replicates in each treatment group listed in S3 Table.

### β-Hydroxybutyrate (β-HBA) measurement and fatty acid oxidation

Male db/db mice were fasted for 3h followed by PO administration of vehicle or compounds. One hour after dosing, stable isotope tracer (^33^D-oleate) formulated with intralipid was intravenously dosed at 50 mg/kg. Plasma was collected at 4h post tracer dosing, using EDTA-coated tubes and centrifuged at 8,500 rpm for 10 min (4C). Plasma was used for β-HBA measurement using the Hydroxybutyrate Color Kit according to the manufacturer’s instructions (BioVision Inc, Milpitas, CA). The levels of plasma ^2^H_2_O were measured using liquid chromatography–mass spectrometry (LC-MS) and ^2^H_2_O generated after compound treatment was used as the surrogate marker for whole body FAO (n = 8).

### Metabolic rate studies

Energy intake and expenditure were measured by indirect calorimetry using an OxyMax system (Columbus Instruments, Columbus, OH) in lean C57BL/6 mice (Taconic), n = 8 per group, fed a regular chow diet. The mice were randomized, placed in individual boxes inside an OxyMax temperature controlled chamber and allowed to acclimate to room temperature for seven days prior study. Energy Expenditure (EE), Respiratory Quotient (RQ)/Respiratory Exchange Ratio (RER), Food Intake (FI), oxygen consumption (VO_2_) and CO_2_ production (VCO_2_), total and ambulatory locomotor activity were recorded in 30 min intervals. RQ/RER was calculated as VCO_2_/VO_2_. Locomotor activity was determined within the OxyMax caging system using an x/y/z-axis infrared light beam system. Total activity was expressed as one count per 2 consecutive beam breaks. Body weight was measured manually on a daily basis. Pair feeding was initiated via blockage of access to food in 10 min intervals. Body composition was assessed by NMR analysis (EchoMRI). SA1 was dosed 2h post-light cycle on QD, PO at 5 ml/kg for 5 days. Data were collected continuously during the treatments.

### Statistics

All data except gene profiling results were plotted by using Graphpad Prism 7.0 (Graphpad Software Inc., La Jolla, CA). Statistical analysis was performed in Graphpad Prism 7.0 by using ANOVA and multiple comparisons. Asterisks denote statistical significance, *p < 0.05, **p < 0. 01, *** p < 0.001 relative to vehicle.

Gene expression data were processed using the Robust Multiarray Average (RMA) pipeline and one-way ANOVA analysis was performed to obtain fold change, raw p values, and False Discovery Rate Benjamini & Hochberg (FDR_BH) corrected p values using the Array Studio Software (Omicsoft Corporation, Cary, NC). Probe sets had to pass a pre-filter of Affymetrix MAS5 present call p-value < 0.05 in > 50% of the samples, per treatment group, to qualify for further analysis. Average fold changes between 1) exercise and sedentary (vehicle treated) or 2) compound and vehicle treatment groups (both sedentary) were generated with the sedentary vehicle group serving as baseline. Box plots, scatter plots and heatmaps were generated using the Array Studio Software (Omicsoft Corporation, Cary, NC). For the heatmap visualizations, individual replicates from all treatment groups (including the sedentary vehicle treated group) were compared to the pooled sedentary vehicle treated group in order to generate individual ratio data.

## Supporting information

S1 Fig Plasma and heart levels of long chain acylcarnitines in DIO model. Levels of long chain acylcarnitines in plasma (A-C) or heart tissue (D-G) after 28 day treatment with SA2 (3 mg/kg, qd, PO).
(PDF)

S2 Fig Effect of AMPK activators on respiratory quotient/exchange ratio and food intake in C57BL/6 mice. Respiratory quotient (A) or cumulative food intake (B) in mice after treatment with vehicle or either LA1 (A) or SA1 (B) at 3, 10, or 30 mg/kg (QD, PO) or mice pair-fed to the LA1 or SA1 group at the indicated times.
(PDF)

S3 Fig Acute exercise and pharmacological AMPK activation have similar transcriptional effects in skeletal muscle. Shown in the scatter plot are the log2 Fold Change values for the 789 probesets that met the +/- 1.2 fold change and FDR_BH p<0.1 threshold in both the acute exercise and acute LA2 (high dose) groups. The corresponding heatmap is shown in Fig 3D and list of probesets in S5 Table.
(PDF)

S4 Fig Acute exercise-specific transcriptional effects in skeletal muscle. Shown in the heat map are the 62 probesets that met the +/- 1.5 fold change and FDR_BH p<0.1 threshold in the acute exercise group (red arrow), and not significantly changed by LA2 and SA2 treatment (both high dose, and both with < +/- 1.2 fold change and FDR_BH p>0.2; black arrows). The color gradient represents fold change compared to vehicle treated sedentary mice (-2.0 to 2.0 fold). The 62 probesets shown here are listed in S6 Table.
(PDF)

S5 Fig Acute pharmacological AMPK activation-specific transcriptional effects in skeletal muscle. Shown in the heat map are the 57 probesets that were significantly regulated by LA2 (+/- 1.5 fold change and FDR_BH p<0.1) and SA2 (+/- 1.2 fold change and FDR_BH p<0.1) (red arrows), and not significantly changed by acute exercise (< +/- 1.2 fold change and FDR_BH p>0.2; black arrow). The color gradient represents fold change compared to vehicle treated sedentary mice (-2.0 to 2.0 fold). The 57 probesets shown here are listed in S7 Table.
(PDF)

S6 Fig Acute pharmacological LA2-specific transcriptional effects in skeletal muscle. Shown in the heat map are the 233 probesets that were significantly regulated by LA2 (+/- 1.5 fold change and FDR_BH p<0.1; red arrow) and not significantly changed by either SA2 or by acute exercise (< +/- 1.2 fold change and FDR_BH p>0.2) (black arrows).The color gradient represents fold change compared to vehicle treated sedentary mice (-2.0 to 2.0 fold). The 233 probesets shown here are listed in S8 Table.
(PDF)

S7 Fig Common transcriptional effects after acute exercise or acute pharmacological AMPK activation in brown adipose tissue (BAT). Shown in the heat map are the 255 probesets that met the +/- 1.2 fold change and FDR_BH p<0.1 threshold in the acute exercise group and acute LA2 (high dose) treatment group (red arrows). The color gradient represents fold change compared to vehicle treated sedentary mice (-2.0 to 2.0 fold). The 255 probesets shown here are listed in S9 Table.
(PDF)

S8 Fig Acute exercise-specific transcriptional effects in brown adipose tissue (BAT). Shown in the heat map are the 26 probesets that met the +/- 1.5 fold change and FDR_BH p<0.1 threshold in the acute exercise group (red arrow), and not significantly changed by LA2 and SA2 treatment (both high dose, and both with < +/- 1.2 fold change and FDR_BH p>0.2; black arrows). The color gradient represents fold change compared to vehicle treated sedentary mice (-2.0 to 2.0 fold). The 26 probesets shown here are listed in S10 Table.
(PDF)

S9 Fig Acute pharmacological AMPK activation-specific transcriptional effects in brown adipose tissue (BAT). Shown in the heat map are the 11 probesets that were significantly regulated by LA2 (+/- 1.5 fold change and FDR_BH p<0.1) and SA2 (+/- 1.2 fold change and FDR_BH p<0.1) (red arrows), and not significantly changed by acute exercise (< +/- 1.2 fold change and FDR_BH p>0.2; black arrow). The color gradient represents fold change compared to vehicle treated sedentary mice (-2.0 to 2.0 fold). The 11 probesets shown here are listed in S11 Table.
(PDF)

S10 Fig Acute pharmacological LA2-specific transcriptional effects in brown adipose tissues (BAT). Shown in the heat map are the 96 probesets that were significantly regulated by LA2 (+/- 1.5 fold change and FDR_BH p<0.1; red arrow) and not significantly changed by either SA2 or by acute exercise (< +/- 1.2 fold change and FDR_BH p>0.2) (black arrows).The color gradient represents fold change compared to vehicle treated sedentary mice (-2.0 to 2.0 fold). The 96 probesets shown here are listed in S12 Table.
(PDF)

S11 Fig Common transcriptional effects after acute exercise or acute pharmacological AMPK activation in heart. Shown in the heat map are the 1072 probesets that met the +/- 1.2 fold change and FDR_BH p<0.1 threshold in the acute exercise group and acute LA2 (high dose) treatment group (red arrows). The color gradient represents fold change compared to vehicle treated sedentary mice (-2.0 to 2.0 fold). The 1072 probesets shown here are listed in S13 Table.
(PDF)

S12 Fig Acute exercise-specific transcriptional effects in heart. Shown in the heat map are the 30 probesets that met the +/- 1.5 fold change and FDR_BH p<0.1 threshold in the acute exercise group (red arrow), and not significantly changed by LA2 and SA2 treatment (both high dose, and both with < +/- 1.2 fold change and FDR_BH p>0.2; black arrows). The color gradient represents fold change compared to vehicle treated sedentary mice (-2.0 to 2.0 fold). The 30 probesets shown here are listed in S14 Table.
(PDF)

S13 Fig Acute pharmacological AMPK activation-specific transcriptional effects in heart. Shown in the heat map are the 61 probesets that were significantly regulated by LA2 (+/- 1.5 fold change and FDR_BH p<0.1) and SA2 (+/- 1.2 fold change and FDR_BH p<0.1) (red arrows), and not significantly changed by acute exercise (< +/- 1.2 fold change and FDR_BH p>0.2; black arrow). The color gradient represents fold change compared to vehicle treated sedentary mice (-2.0 to 2.0 fold). The 61 probesets shown here are listed in S15 Table.
(PDF)

S14 Fig Acute pharmacological LA2-specific transcriptional effects in heart. Shown in the heat map are the 175 probesets that were significantly regulated by LA2 (+/- 1.5 fold change and FDR_BH p<0.1; red arrow) and not significantly changed by either SA2 or by acute exercise (< +/- 1.2 fold change and FDR_BH p>0.2) (black arrows).The color gradient represents fold change compared to vehicle treated sedentary mice (-2.0 to 2.0 fold). The 175 probesets shown here are listed in S16 Table.
(PDF)

S15 Fig Common transcriptional effects after acute exercise or acute pharmacological AMPK activation in inguinal white adipose tissue (IWAT). Shown in the heat map are the 118 probesets that met the +/- 1.2 fold change and FDR_BH p<0.1 threshold in the acute exercise group and acute LA2 (high dose) treatment group (red arrows). The color gradient represents fold change compared to vehicle treated sedentary mice (-2.0 to 2.0 fold). The 118 probesets shown here are listed in S17 Table.
(PDF)

S16 Fig Acute exercise-specific transcriptional effects in inguinal white adipose tissue (IWAT). Shown in the heat map are the 23 probesets that met the +/- 1.5 fold change and FDR_BH p<0.1 threshold in the acute exercise group (red arrow), and not significantly changed by LA2 and SA2 treatment (both high dose, and both with < +/- 1.2 fold change and FDR_BH p>0.2; black arrows). The color gradient represents fold change compared to vehicle treated sedentary mice (-2.0 to 2.0 fold). The 23 probesets shown here are listed in S18 Table.
(PDF)

S17 Fig Acute pharmacological LA2-specific transcriptional effects in inguinal white adipose tissue (IWAT). Shown in the heat map are the 165 probesets that were significantly regulated by LA2 (+/- 1.5 fold change and FDR_BH p<0.1; red arrow) and not significantly changed by either SA2 or by acute exercise (< +/- 1.2 fold change and FDR_BH p>0.2) (black arrows).The color gradient represents fold change compared to vehicle treated sedentary mice (-2.0 to 2.0 fold). The 165 probesets shown here are listed in S19 Table.
(PDF)

S18 Fig Common transcriptional effects after acute exercise or acute pharmacological AMPK activation in liver. Shown in the heat map are the 710 probesets that met the +/- 1.2 fold change and FDR_BH p<0.1 threshold in the acute exercise group and acute LA2 (high dose) treatment group (red arrows). The color gradient represents fold change compared to vehicle treated sedentary mice (-2.0 to 2.0 fold). The 710 probesets shown here are listed in S20 Table.
(PDF)

S19 Fig Acute exercise-specific transcriptional effects in liver. Shown in the heat map are the 59 probesets that met the +/- 1.5 fold change and FDR_BH p<0.1 threshold in the acute exercise group (red arrow), and not significantly changed by LA2 and SA2 treatment (both high dose, and both with < +/- 1.2 fold change and FDR_BH p>0.2; black arrows). The color gradient represents fold change compared to vehicle treated sedentary mice (-2.0 to 2.0 fold). The 59 probesets shown here are listed in S21 Table.
(PDF)

S20 Fig Acute pharmacological AMPK activation-specific transcriptional effects in liver. Shown in the heat map are the 13 probesets that were significantly regulated by LA2 (+/- 1.5 fold change and FDR_BH p<0.1) and SA2 (+/- 1.2 fold change and FDR_BH p<0.1) (red arrows), and not significantly changed by acute exercise (< +/- 1.2 fold change and FDR_BH p>0.2; black arrow). The color gradient represents fold change compared to vehicle treated sedentary mice (-2.0 to 2.0 fold). The 13 probesets shown here are listed in S22 Table.
(PDF)

S21 Fig Acute pharmacological LA2-specific transcriptional effects in liver. Shown in the heat map are the 243 probesets that were significantly regulated by LA2 (+/- 1.5 fold change and FDR_BH p<0.1; red arrow) and not significantly changed by either SA2 or by acute exercise (< +/- 1.2 fold change and FDR_BH p>0.2) (black arrows).The color gradient represents fold change compared to vehicle treated sedentary mice (-2.0 to 2.0 fold). The 243 probesets shown here are listed in S23 Table.
(PDF)

S22 Fig Zbtb16 was one of six genes (represented by 7 probesets) that were significantly regulated (+/- 1.2 fold change with FDR_BH <0.1) by both acute exercise and acute pharmacological AMPK activation (LA2, high dose) in all 5 tissues profiled. Shown in the box plot are the log2 Intensity values per treatment group.
(PDF)

S23 Fig Bcl2l1 was one of six genes (represented by 7 probesets) that were significantly regulated (+/- 1.2 fold change with FDR_BH <0.1) by both acute exercise and acute pharmacological AMPK activation (LA2, high dose) in all 5 tissues profiled. Shown in the box plot are the log2 Intensity values per treatment group.
(PDF)

S24 Fig Map3k6 was one of six genes (represented by 7 probesets) that were significantly regulated (+/- 1.2 fold change with FDR_BH <0.1) by both acute exercise and acute pharmacological AMPK activation (LA2, high dose) in all 5 tissues profiled. Shown in the box plot are the log2 Intensity values per treatment group.
(PDF)

S25 Fig Cdkn1a was one of six genes (represented by 7 probesets) that were significantly regulated (+/- 1.2 fold change with FDR_BH <0.1) by both acute exercise and acute pharmacological AMPK activation (LA2, high dose) in all 5 tissues profiled. Shown in the box plot are the log2 Intensity values per treatment group.
(PDF)

S26 Fig BC031353 was one of six genes (represented by 7 probesets) that were significantly regulated (+/- 1.2 fold change with FDR_BH <0.1) by both acute exercise and acute pharmacological AMPK activation (LA2, high dose) in all 5 tissues profiled. Shown in the box plot are the log2 Intensity values per treatment group.
(PDF)

S27 Fig Cdkn1a was one of six genes (represented by 7 probesets) that were significantly regulated (+/- 1.2 fold change with FDR_BH <0.1) by both acute exercise and acute pharmacological AMPK activation (LA2, high dose) in all 5 tissues profiled. Shown in the box plot are the log2 Intensity values per treatment group.
(PDF)

S28 Fig Regulation of genes involved in glycogen, glucose, and lipid metabolism specifically by LA1. Shown in the box plots are the log2_Intensity values per treatment group for genes involved in glycogen, glucose, and lipid metabolism. See S24 Table for the corresponding data.
(PDF)

S29 Fig Cardiac AMPK activation by acute LA1 treatment. pACC/ACC ratio in the heart of male db/+ mice after 2 hour of treatment of LA1 at 10 and 30 mpk (PO, n = 6).
(PDF)

S30 Fig Chronic effect of LA1 and SA2 on liver and plasma lipid in DIO mice. After 12 weeks on high fat diet, the animals were treated by oral administration of LA1 at 3 mg/kg per day for 21 days (A) or SA2 at 3 mpk for 30 days (B–D) (n = 10). Hepatic lipids were measured using magnetic resonance imaging and proton magnetic resonance spectroscopy (1H-MRS) at the end of the study. “Liver TG (% change)” in B is the % change from baseline of the mice after treatment. These mice were imaged prior to treatment, and the treatment effect was calculated based on the TG changes of each individual mouse compared with its own baseline. Plasma triglyceride (TG) and apolipoprotein B (ApoB) were measured by Infinity Triglycerides Reagent (Thermo Scientific) and mouse ApoB ELISA kit (Abcam), respectively.
(PDF)

S1 Table Compounds EC50 values. EC50 values and %Max activation (relative to the maximal activation induced by AMP), for mouse enzymes.
(XLSX)

S2 Table Affymetrix chip IDs. (XLSX)

S3 Table Number of replicate samples for RNA profiling. (XLSX)

S4 Table Signature counts. (XLSX)

S5 Table Vastus muscle intersection signature. 789 probesets significantly altered by both acute exercise and acute LA2 treatment in vastus lateralis (at least 1.2-fold change and FDR_BH <0.1). See corresponding heat map in Fig 3D and scatter plot in S3 Fig.
(XLSX)

S6 Table Vastus muscle exercise-specific signature. 62 probesets robustly regulated only by acute exercise, and not by acute AMPK activator treatment, in vastus lateralis (at least 1.5-fold change and FDR_BH <0.1). See corresponding heat map in S4 Fig.
(XLSX)

S7 Table Vastus muscle AMPK activator-specific signature. 57 probesets significantly regulated only after acute AMPK activator treatment (at least 1.5-fold change and FDR_BH <0.1 by LA2, and at least 1.2 fold change and FDR_BH p<0.1 by SA2), and not by acute exercise, in vastus lateralis. See corresponding heat map in S5 Fig.
(XLSX)

S8 Table Vastus muscle LA2-specific signature. 233 probesets significantly regulated only after acute LA2 treatment (at least 1.5-fold change and FDR_BH <0.1 by LA2, and not significantly changed by either SA2 or by acute exercise (< +/- 1.2 fold change and FDR_BH p>0.2) in vastus lateralis. See corresponding heat map in S6 Fig.
(XLSX)

S9 Table BAT intersection signature. 255 probesets significantly altered by both acute exercise and acute LA2 treatment in BAT (at least 1.2-fold change and FDR_BH <0.1). See corresponding heat map in S7 Fig.
(XLSX)

S10 Table BAT exercise-specific signature. 26 probesets robustly regulated only by acute exercise, and not by acute AMPK activator treatment, in BAT (at least 1.5-fold change and FDR_BH <0.1). See corresponding heat map in S8 Fig.
(XLSX)

S11 Table BAT AMPK activator-specific signature. 11 probesets significantly regulated only after acute AMPK activator treatment (at least 1.5-fold change and FDR_BH <0.1 by LA2, and at least 1.2 fold change and FDR_BH p<0.1 by SA2), and not by acute exercise, in BAT. See corresponding heat map in S9 Fig.
(XLSX)

S12 Table BAT LA2-specific signature. 96 probesets significantly regulated only after acute LA2 treatment (at least 1.5-fold change and FDR_BH <0.1 by LA2, and not significantly changed by either SA2 or by acute exercise (< +/- 1.2 fold change and FDR_BH p>0.2) in BAT. See corresponding heat map in S10 Fig.
(XLSX)

S13 Table Heart intersection signature. 1072 probesets significantly altered by both acute exercise and acute LA2 treatment in heart (at least 1.2-fold change and FDR_BH <0.1). See corresponding heat map in Fig 3D and scatter plot in S11 Fig.
(XLSX)

S14 Table Heart exercise-specific signature. 30 probesets robustly regulated only by acute exercise, and not by acute AMPK activator treatment, in heart (at least 1.5-fold change and FDR_BH <0.1). See corresponding heat map in S12 Fig.
(XLSX)

S15 Table Heart AMPK activator-specific signature. 61 probesets significantly regulated only after acute AMPK activator treatment (at least 1.5-fold change and FDR_BH <0.1 by LA2, and at least 1.2 fold change and FDR_BH p<0.1 by SA2), and not by acute exercise, in heart. See corresponding heat map in S13 Fig.
(XLSX)

S16 Table Heart LA2-specific signature. 175 probesets significantly regulated only after acute LA2 treatment (at least 1.5-fold change and FDR_BH <0.1 by LA2, and not significantly changed by either SA2 or by acute exercise (< +/- 1.2 fold change and FDR_BH p>0.2) in heart. See corresponding heat map in S14 Fig.
(XLSX)

S17 Table IWAT intersection signature. 118 probesets significantly altered by both acute exercise and acute LA2 treatment in IWAT (at least 1.2-fold change and FDR_BH <0.1). See corresponding heat map in Fig 3D and scatter plot in S15 Fig.
(XLSX)

S18 Table IWAT exercise-specific signature. 23 probesets robustly regulated only by acute exercise, and not by acute AMPK activator treatment, in IWAT (at least 1.5-fold change and FDR_BH <0.1). See corresponding heat map in S16 Fig.
(XLSX)

S19 Table IWAT LA2-specific signature. 165 probesets significantly regulated only after acute LA2 treatment (at least 1.5-fold change and FDR_BH <0.1 by LA2, and not significantly changed by either SA2 or by acute exercise (< +/- 1.2 fold change and FDR_BH p>0.2) in IWAT. See corresponding heat map in S17 Fig.
(XLSX)

S20 Table Liver intersection signature. 710 probesets significantly altered by both acute exercise and acute LA2 treatment in liver (at least 1.2-fold change and FDR_BH <0.1). See corresponding heat map in Fig 3D and scatter plot in S18 Fig.
(XLSX)

S21 Table Liver exercise-specific signature. 59 probesets robustly regulated only by acute exercise, and not by acute AMPK activator treatment, in liver (at least 1.5-fold change and FDR_BH <0.1). See corresponding heat map in S19 Fig.
(XLSX)

S22 Table Liver AMPK activator-specific signature. 13 probesets significantly regulated only after acute AMPK activator treatment (at least 1.5-fold change and FDR_BH <0.1 by LA2, and at least 1.2 fold change and FDR_BH p<0.1 by SA2), and not by acute exercise, in liver. See corresponding heat map in S20 Fig.
(XLSX)

S23 Table Vastus muscle LA2-specific signature. 243 probesets significantly regulated only after acute LA2 treatment (at least 1.5-fold change and FDR_BH <0.1 by LA2, and not significantly changed by either SA2 or by acute exercise (< +/- 1.2 fold change and FDR_BH p>0.2) in liver. See corresponding heat map in S21 Fig.
(XLSX)

S24 Table Regulation of genes in glycogen, glucose and fatty acid metabolism pathways. (XLSX)
