# Supplementary figures and images for: Pharmacological AMPK activation induces transcriptional responses congruent to exercise in skeletal and cardiac muscle, adipose tissues and liver
Source: PLoS One. 2019 Feb 27;14(2):e0211568. doi: 10.1371/journal.pone.0211568 (PMC6392219; doi:10.1371/journal.pone.0211568)

**S1 Fig.**

**A**

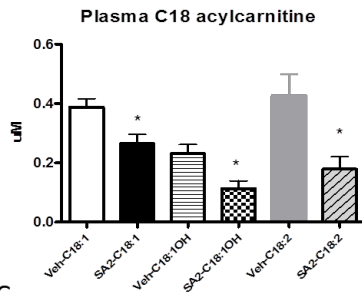

**B**

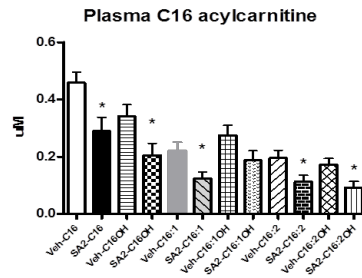

**C**

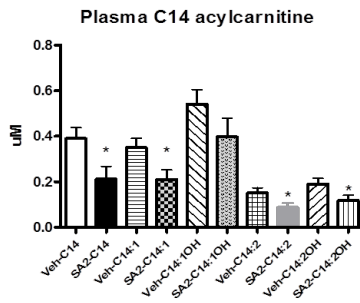

**D**

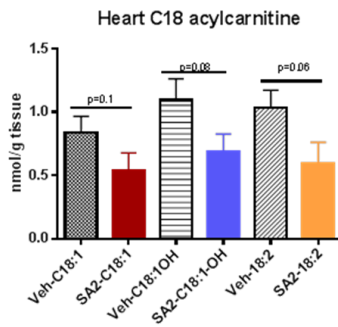

**E**

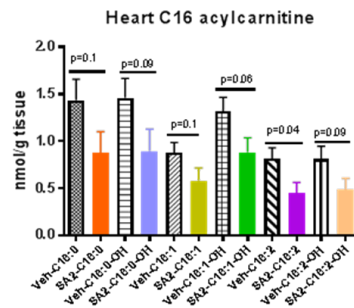

**F**

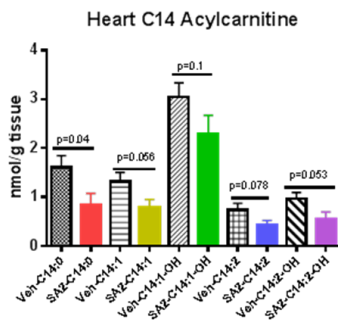

**G**

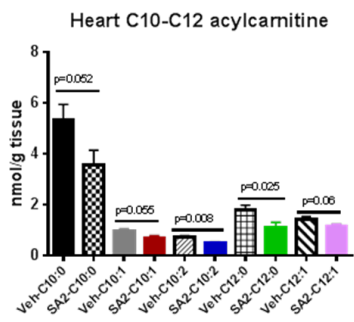

Supplement: S1 Fig — Levels of long chain acylcarnitines in plasma (A-C) or heart tissue (D-G) after 28 day treatment with SA2 (3 mg/kg, qd, PO). (PDF) [file pone.0211568.s001.pdf]

S2 Fig.

A.

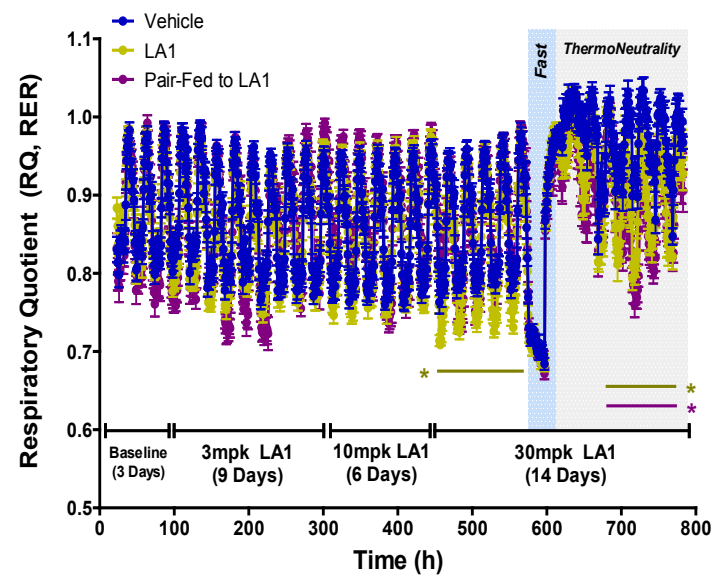

B.

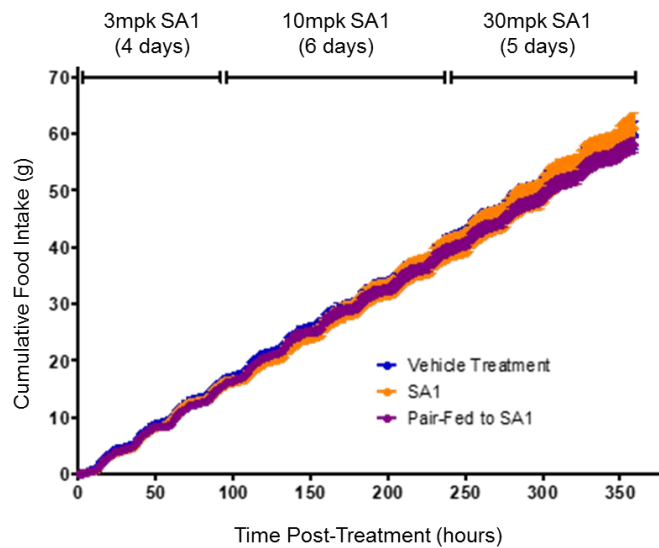

Supplement: S2 Fig — Respiratory quotient (A) or cumulative food intake (B) in mice after treatment with vehicle or either LA1 (A) or SA1 (B) at 3, 10, or 30 mg/kg (QD, PO) or mice pair-fed to the LA1 or SA1 group at the indicated times. (PDF) [file pone.0211568.s002.pdf]

S3 Fig.

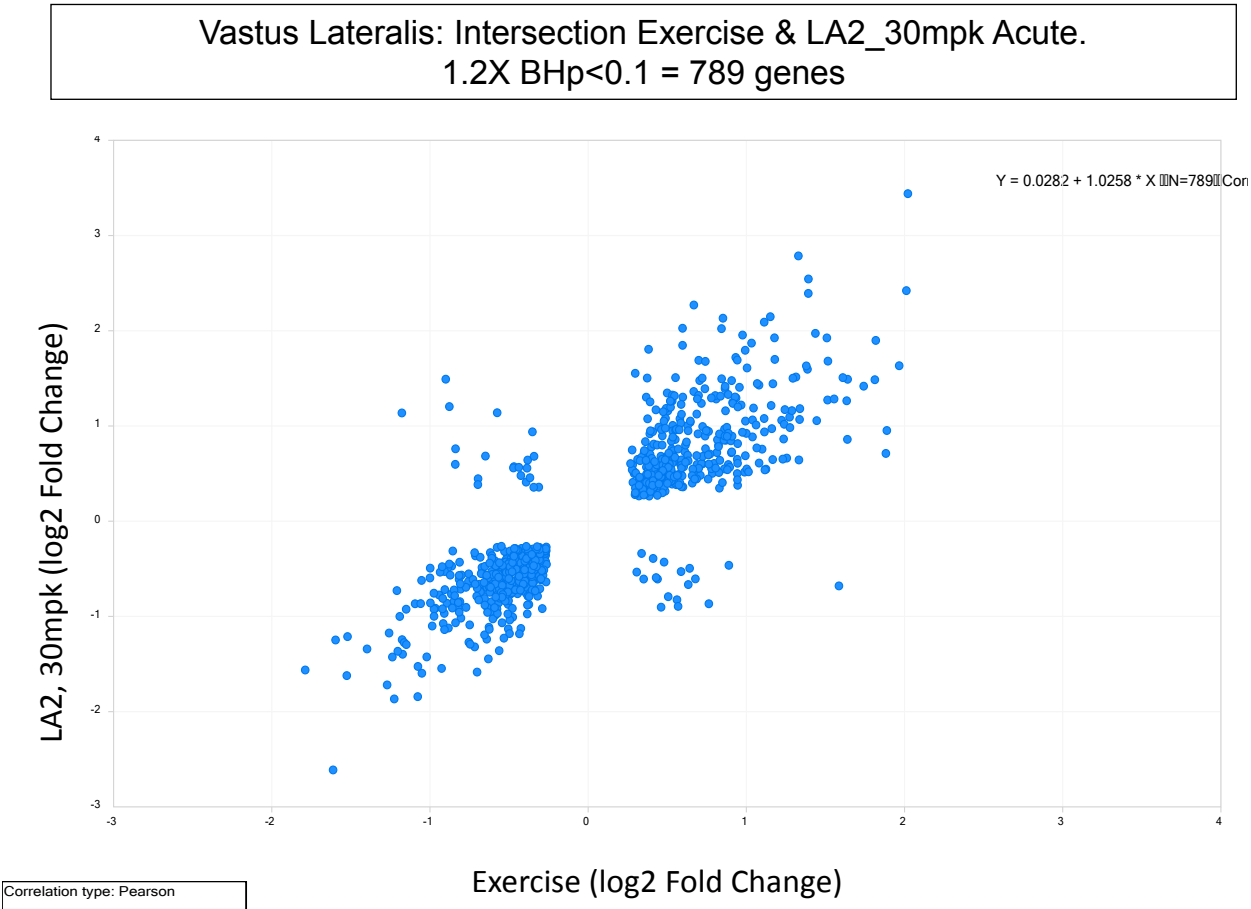

Supplement: S3 Fig — Shown in the scatter plot are the log2 Fold Change values for the 789 probesets that met the +/- 1.2 fold change and FDR_BH p<0.1 threshold in both the acute exercise and acute LA2 (high dose) groups. The corresponding heatmap is shown in Fig 3D and list of probesets in S5 Table. (PDF) [file pone.0211568.s003.pdf]

S4 Fig.

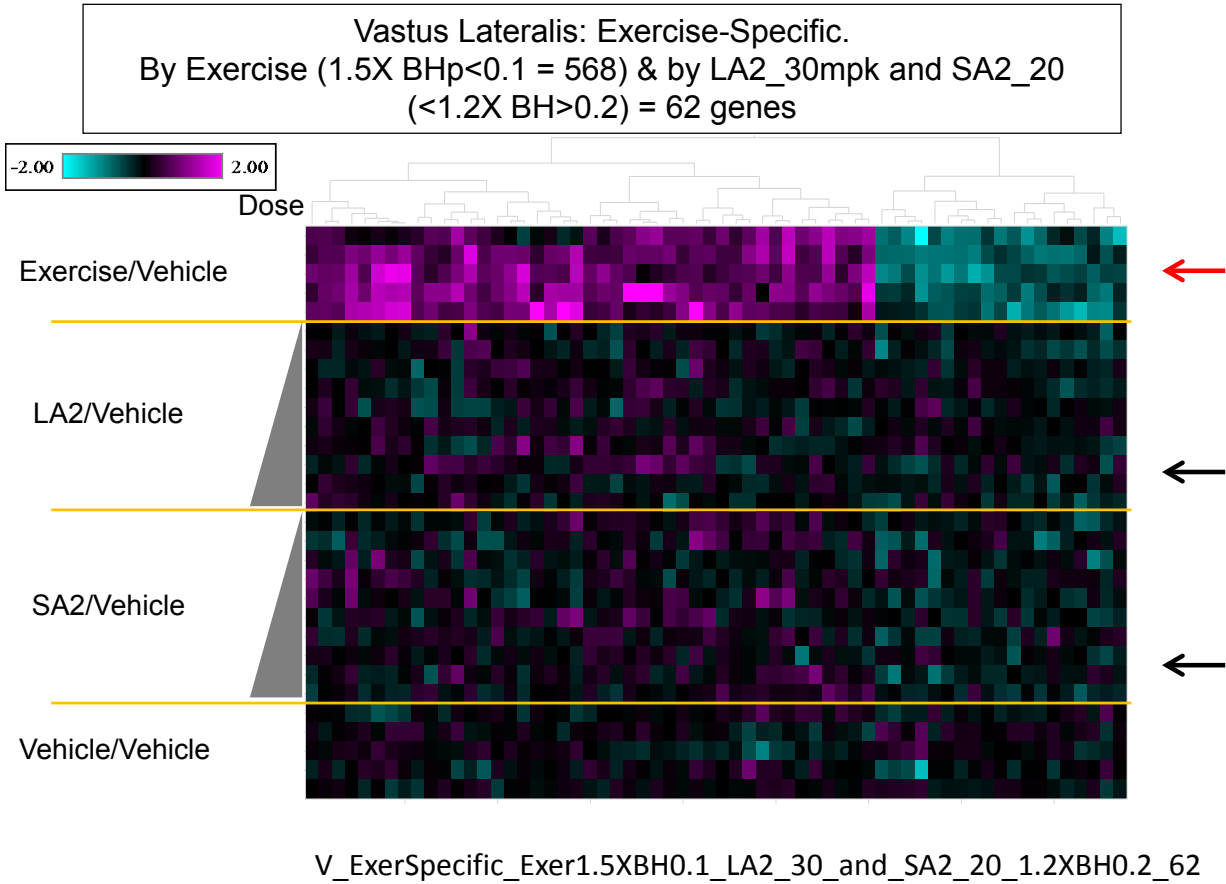

Supplement: S4 Fig — Shown in the heat map are the 62 probesets that met the +/- 1.5 fold change and FDR_BH p<0.1 threshold in the acute exercise group (red arrow), and not significantly changed by LA2 and SA2 treatment (both high dose, and both with < +/- 1.2 fold change and FDR_BH p>0.2; black arrows). The color gradient represents fold change compared to vehicle treated sedentary mice (-2.0 to 2.0 fold). The 62 probesets shown here are listed in S6 Table. (PDF) [file pone.0211568.s004.pdf]

S5 Fig.

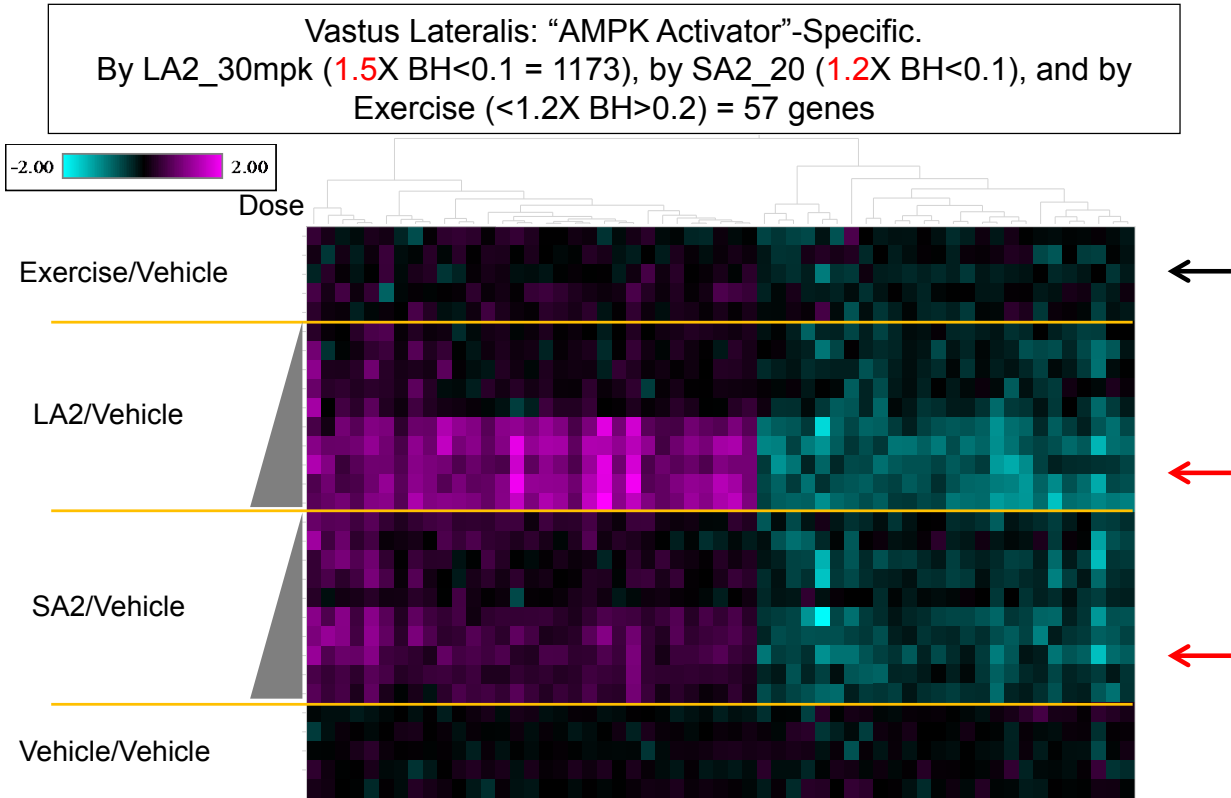

V\_AMPKSpecific\_LA2\_30\_1.5XBH0.1\_SA2\_20\_1.2XBH0.1\_NOT\_Exer\_1.2XBH0.2\_57

Supplement: S5 Fig — Shown in the heat map are the 57 probesets that were significantly regulated by LA2 (+/- 1.5 fold change and FDR_BH p<0.1) and SA2 (+/- 1.2 fold change and FDR_BH p<0.1) (red arrows), and not significantly changed by acute exercise (< +/- 1.2 fold change and FDR_BH p>0.2; black arrow). The color gradient represents fold change compared to vehicle treated sedentary mice (-2.0 to 2.0 fold). The 57 probesets shown here are listed in S7 Table. (PDF) [file pone.0211568.s005.pdf]

S6 Fig.

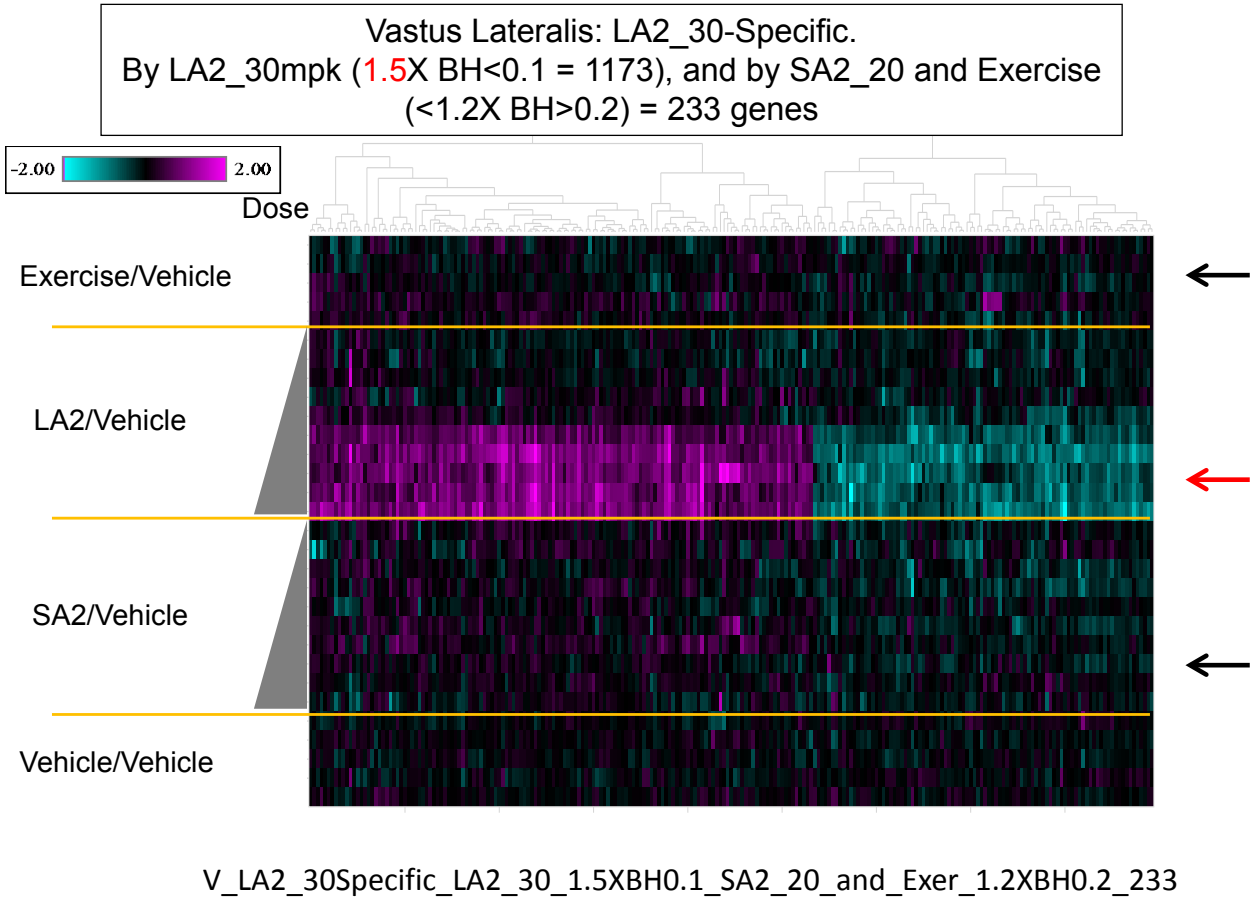

Supplement: S6 Fig — Shown in the heat map are the 233 probesets that were significantly regulated by LA2 (+/- 1.5 fold change and FDR_BH p<0.1; red arrow) and not significantly changed by either SA2 or by acute exercise (< +/- 1.2 fold change and FDR_BH p>0.2) (black arrows).The color gradient represents fold change compared to vehicle treated sedentary mice (-2.0 to 2.0 fold). The 233 probesets shown here are listed in S8 Table. (PDF) [file pone.0211568.s006.pdf]

S7 Fig.

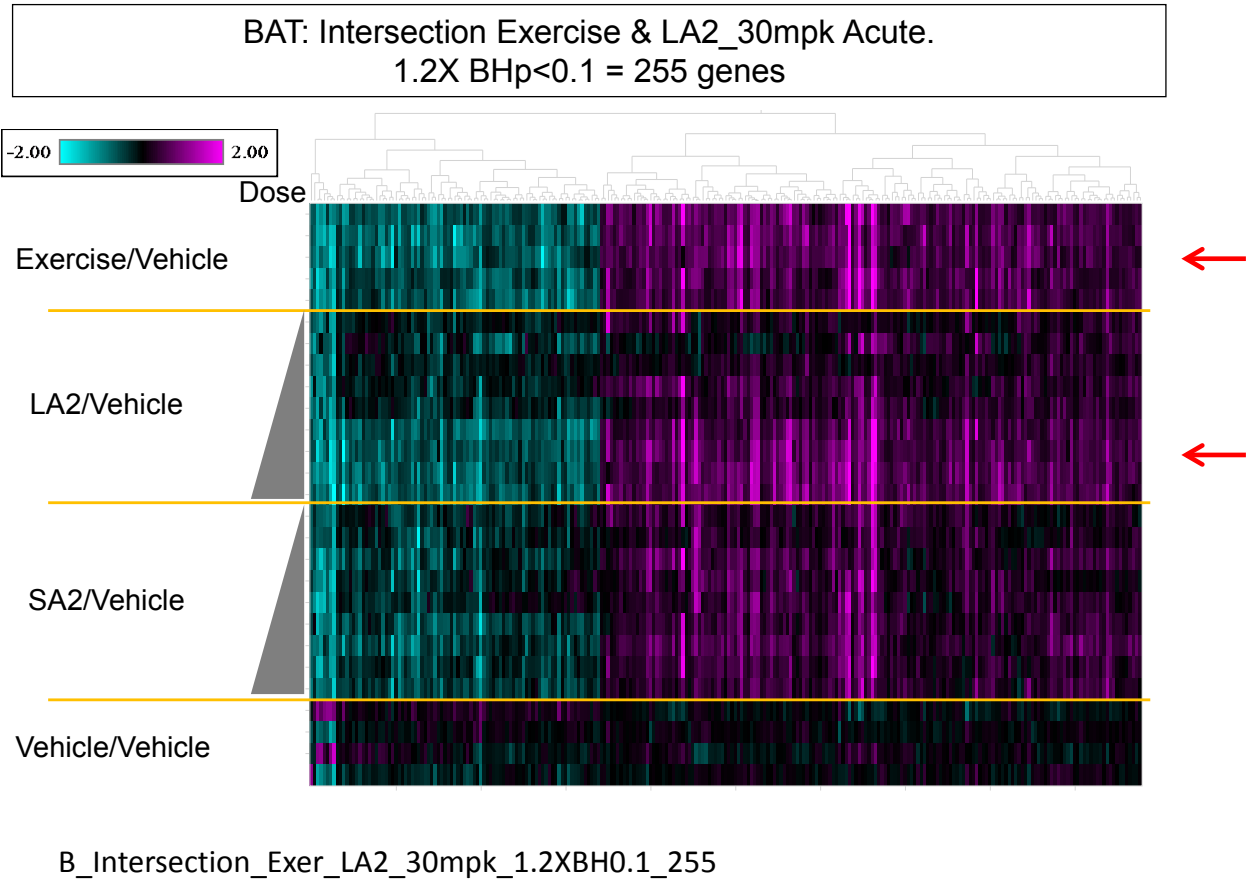

Supplement: S7 Fig — Shown in the heat map are the 255 probesets that met the +/- 1.2 fold change and FDR_BH p<0.1 threshold in the acute exercise group and acute LA2 (high dose) treatment group (red arrows). The color gradient represents fold change compared to vehicle treated sedentary mice (-2.0 to 2.0 fold). The 255 probesets shown here are listed in S9 Table. (PDF) [file pone.0211568.s007.pdf]

S8 Fig.

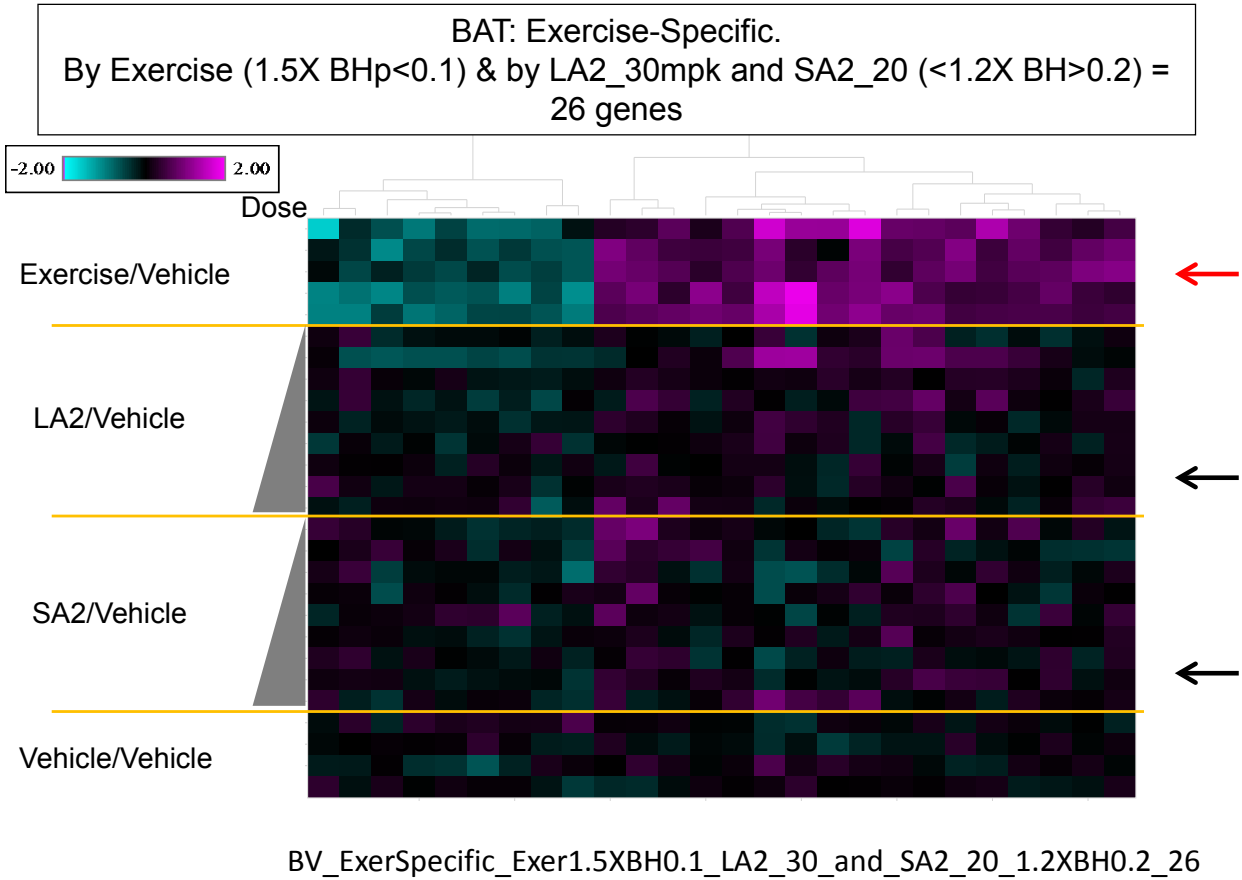

Supplement: S8 Fig — Shown in the heat map are the 26 probesets that met the +/- 1.5 fold change and FDR_BH p<0.1 threshold in the acute exercise group (red arrow), and not significantly changed by LA2 and SA2 treatment (both high dose, and both with < +/- 1.2 fold change and FDR_BH p>0.2; black arrows). The color gradient represents fold change compared to vehicle treated sedentary mice (-2.0 to 2.0 fold). The 26 probesets shown here are listed in S10 Table. (PDF) [file pone.0211568.s008.pdf]

S9 Fig.

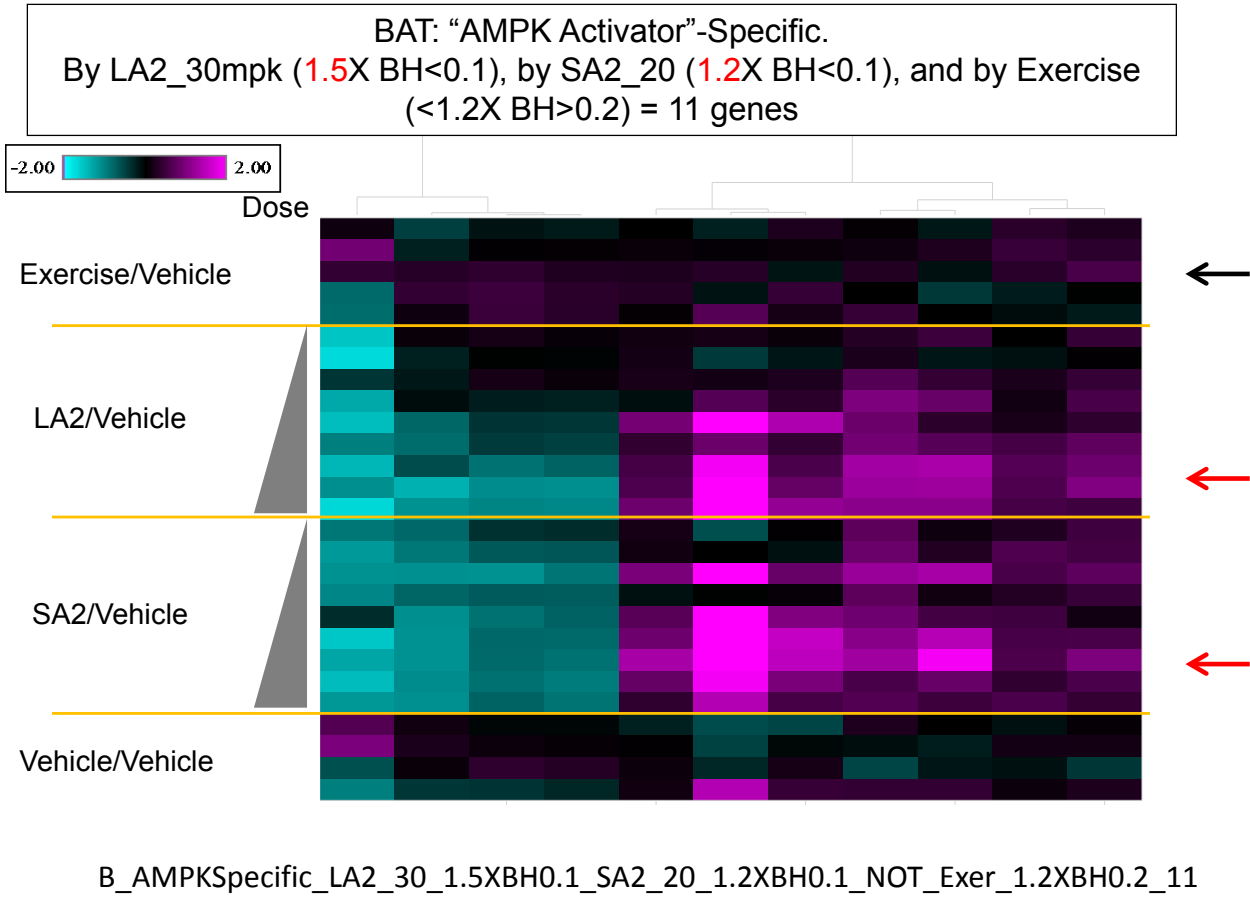

Supplement: S9 Fig — Shown in the heat map are the 11 probesets that were significantly regulated by LA2 (+/- 1.5 fold change and FDR_BH p<0.1) and SA2 (+/- 1.2 fold change and FDR_BH p<0.1) (red arrows), and not significantly changed by acute exercise (< +/- 1.2 fold change and FDR_BH p>0.2; black arrow). The color gradient represents fold change compared to vehicle treated sedentary mice (-2.0 to 2.0 fold). The 11 probesets shown here are listed in S11 Table. (PDF) [file pone.0211568.s009.pdf]

S10 Fig.

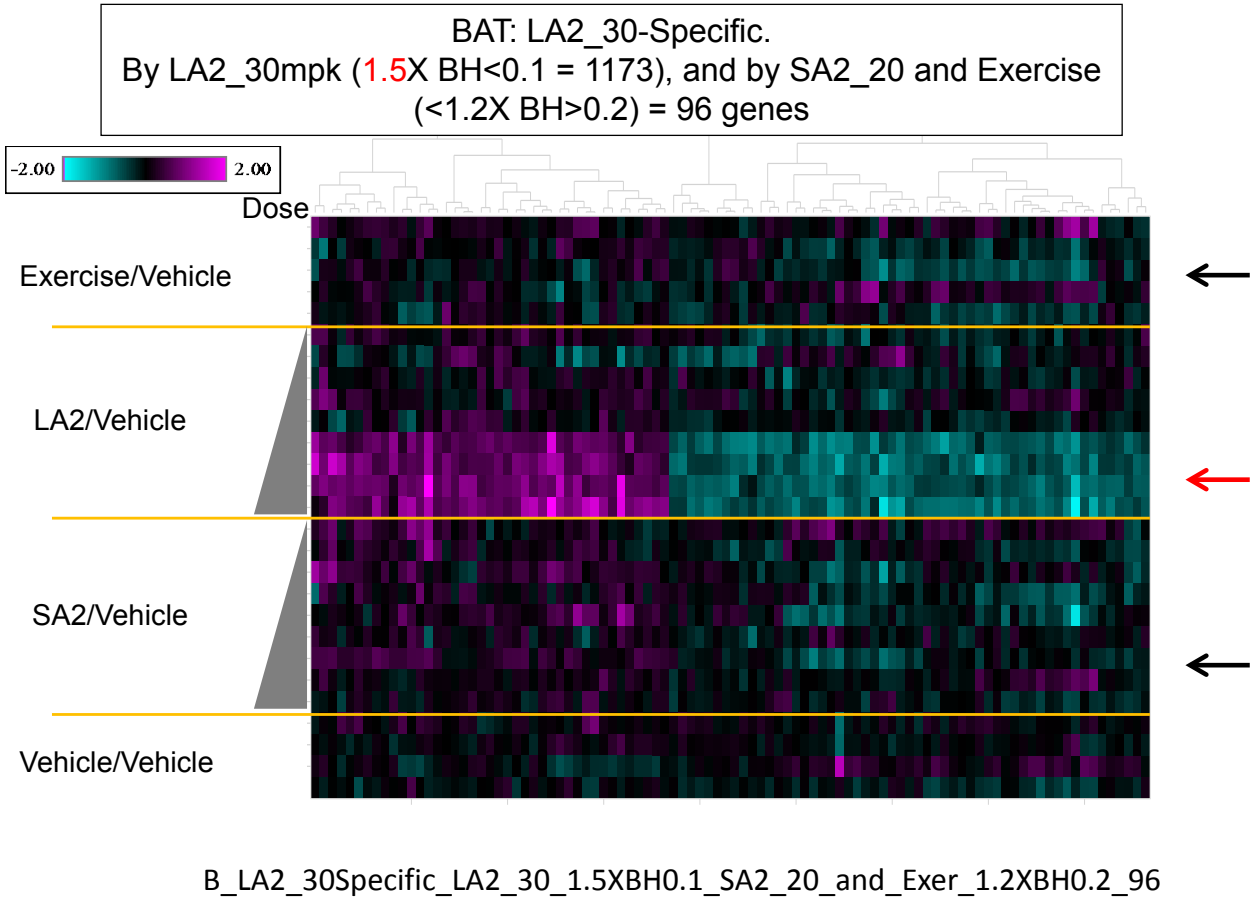

Supplement: S10 Fig — Shown in the heat map are the 96 probesets that were significantly regulated by LA2 (+/- 1.5 fold change and FDR_BH p<0.1; red arrow) and not significantly changed by either SA2 or by acute exercise (< +/- 1.2 fold change and FDR_BH p>0.2) (black arrows).The color gradient represents fold change compared to vehicle treated sedentary mice (-2.0 to 2.0 fold). The 96 probesets shown here are listed in S12 Table. (PDF) [file pone.0211568.s010.pdf]

S11 Fig.

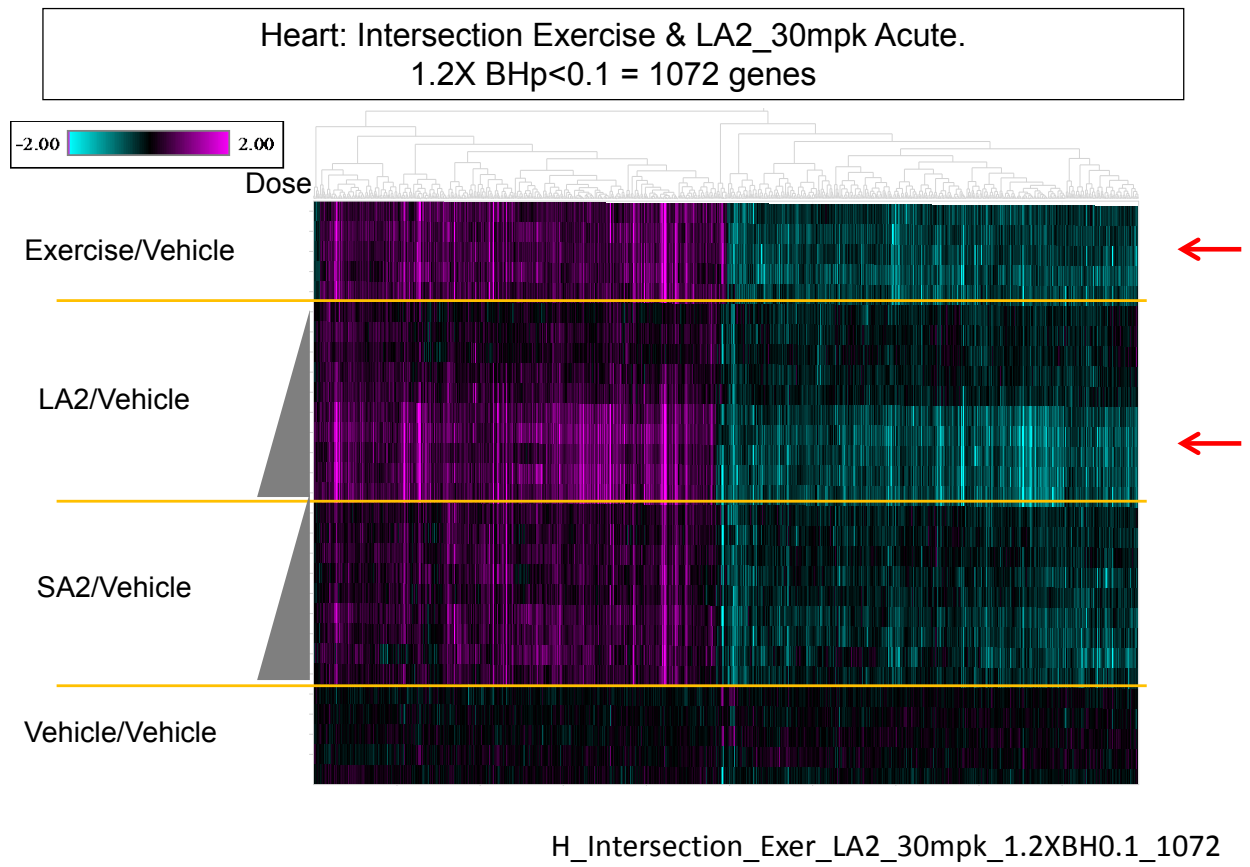

Supplement: S11 Fig — Shown in the heat map are the 1072 probesets that met the +/- 1.2 fold change and FDR_BH p<0.1 threshold in the acute exercise group and acute LA2 (high dose) treatment group (red arrows). The color gradient represents fold change compared to vehicle treated sedentary mice (-2.0 to 2.0 fold). The 1072 probesets shown here are listed in S13 Table. (PDF) [file pone.0211568.s011.pdf]

S12 Fig.

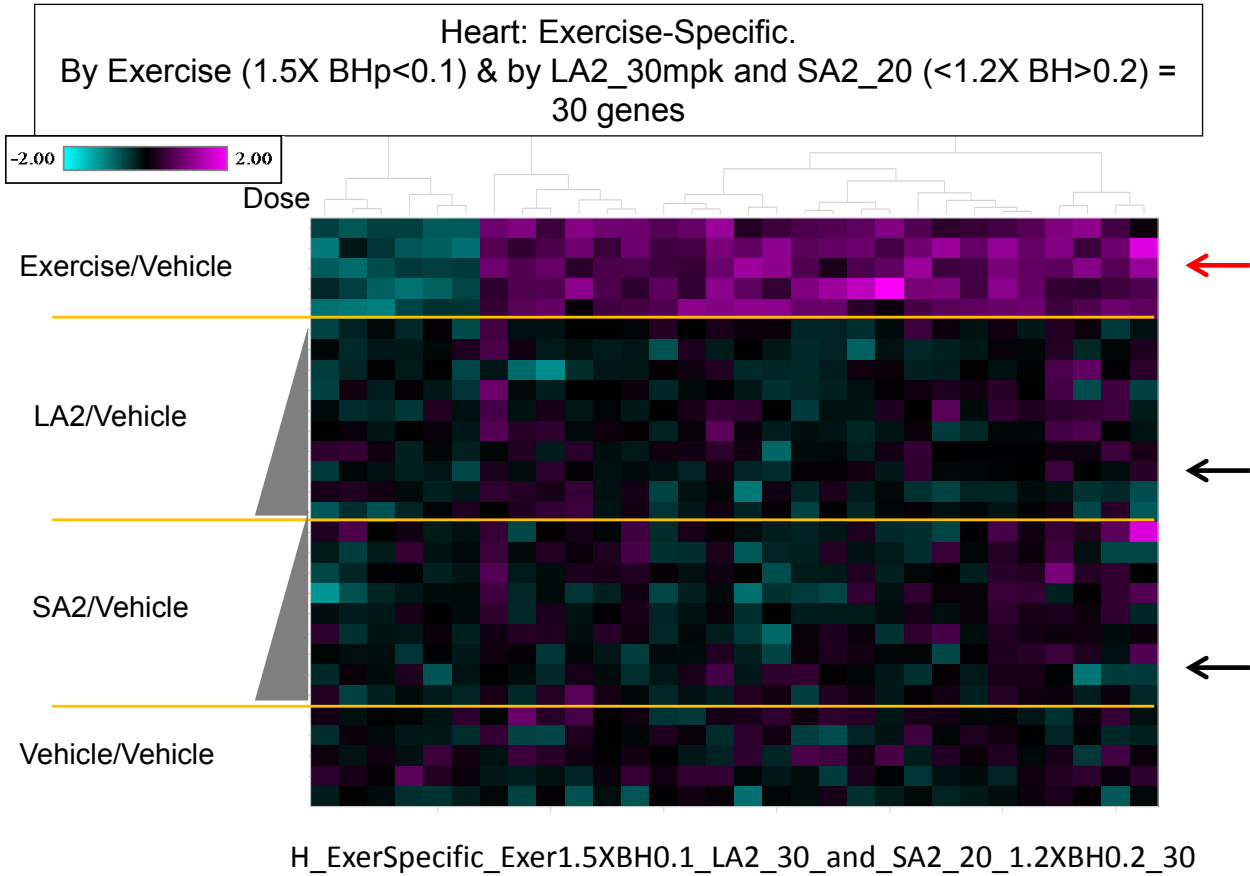

Supplement: S12 Fig — Shown in the heat map are the 30 probesets that met the +/- 1.5 fold change and FDR_BH p<0.1 threshold in the acute exercise group (red arrow), and not significantly changed by LA2 and SA2 treatment (both high dose, and both with < +/- 1.2 fold change and FDR_BH p>0.2; black arrows). The color gradient represents fold change compared to vehicle treated sedentary mice (-2.0 to 2.0 fold). The 30 probesets shown here are listed in S14 Table. (PDF) [file pone.0211568.s012.pdf]

S13 Fig.

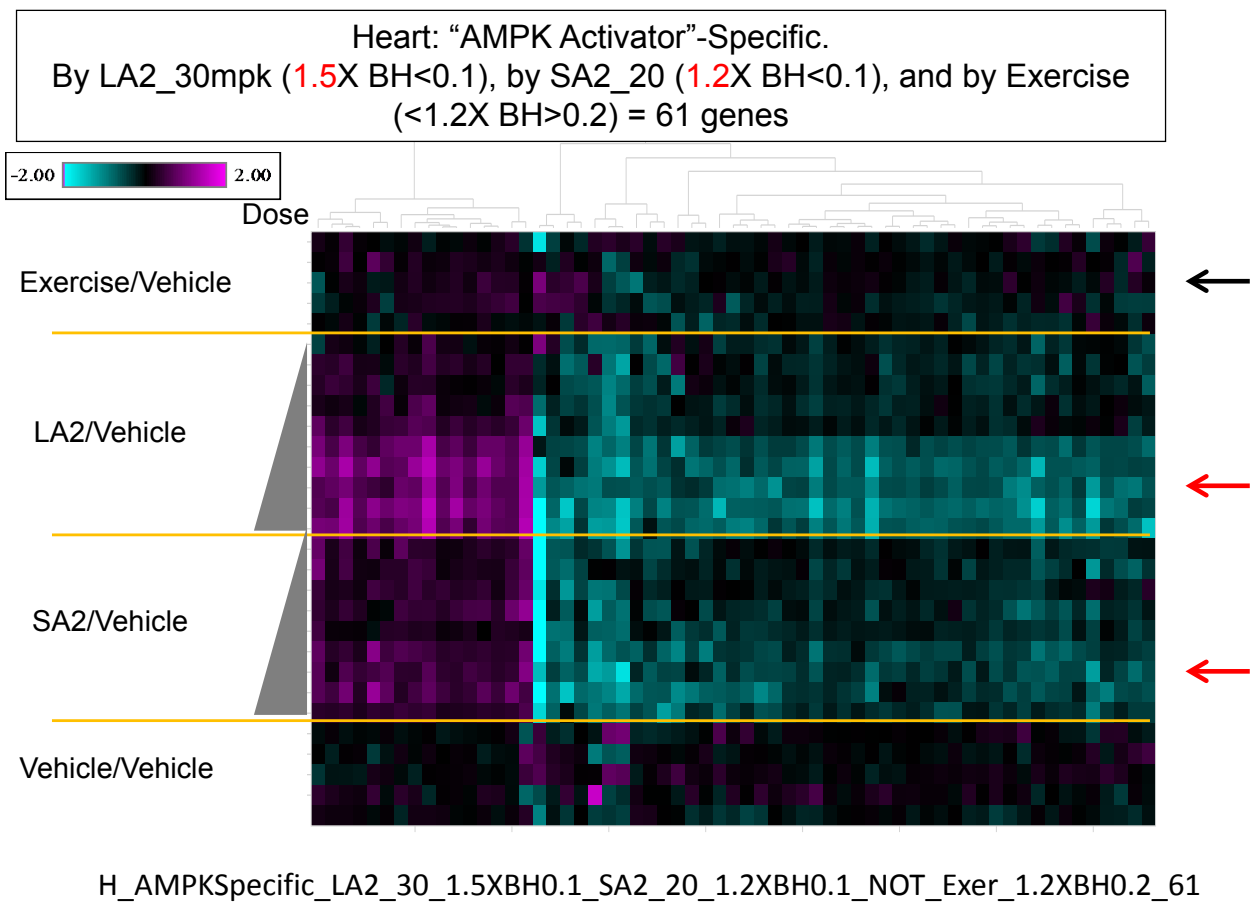

Supplement: S13 Fig — Shown in the heat map are the 61 probesets that were significantly regulated by LA2 (+/- 1.5 fold change and FDR_BH p<0.1) and SA2 (+/- 1.2 fold change and FDR_BH p<0.1) (red arrows), and not significantly changed by acute exercise (< +/- 1.2 fold change and FDR_BH p>0.2; black arrow). The color gradient represents fold change compared to vehicle treated sedentary mice (-2.0 to 2.0 fold). The 61 probesets shown here are listed in S15 Table. (PDF) [file pone.0211568.s013.pdf]

S14 Fig.

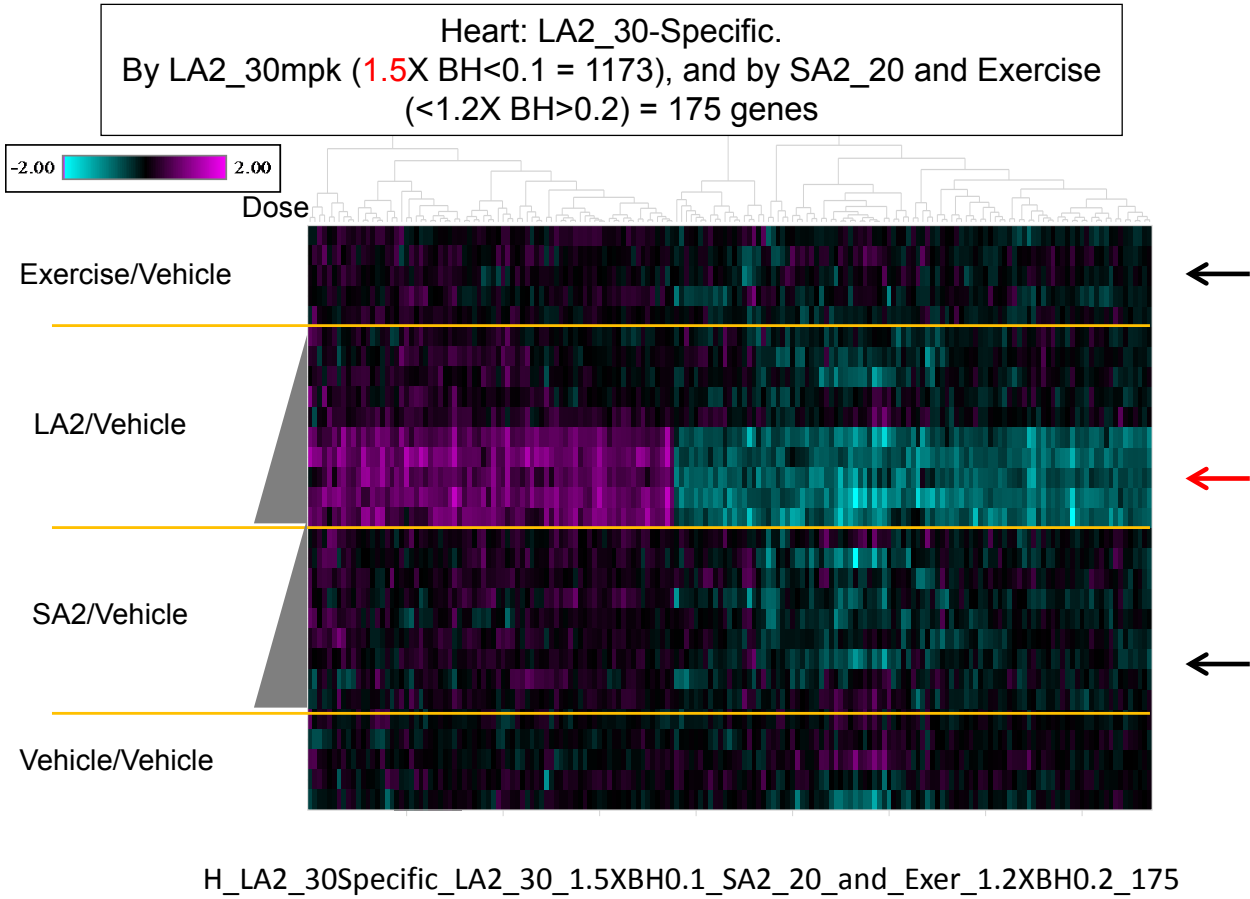

Supplement: S14 Fig — Shown in the heat map are the 175 probesets that were significantly regulated by LA2 (+/- 1.5 fold change and FDR_BH p<0.1; red arrow) and not significantly changed by either SA2 or by acute exercise (< +/- 1.2 fold change and FDR_BH p>0.2) (black arrows).The color gradient represents fold change compared to vehicle treated sedentary mice (-2.0 to 2.0 fold). The 175 probesets shown here are listed in S16 Table. (PDF) [file pone.0211568.s014.pdf]

S15 Fig.

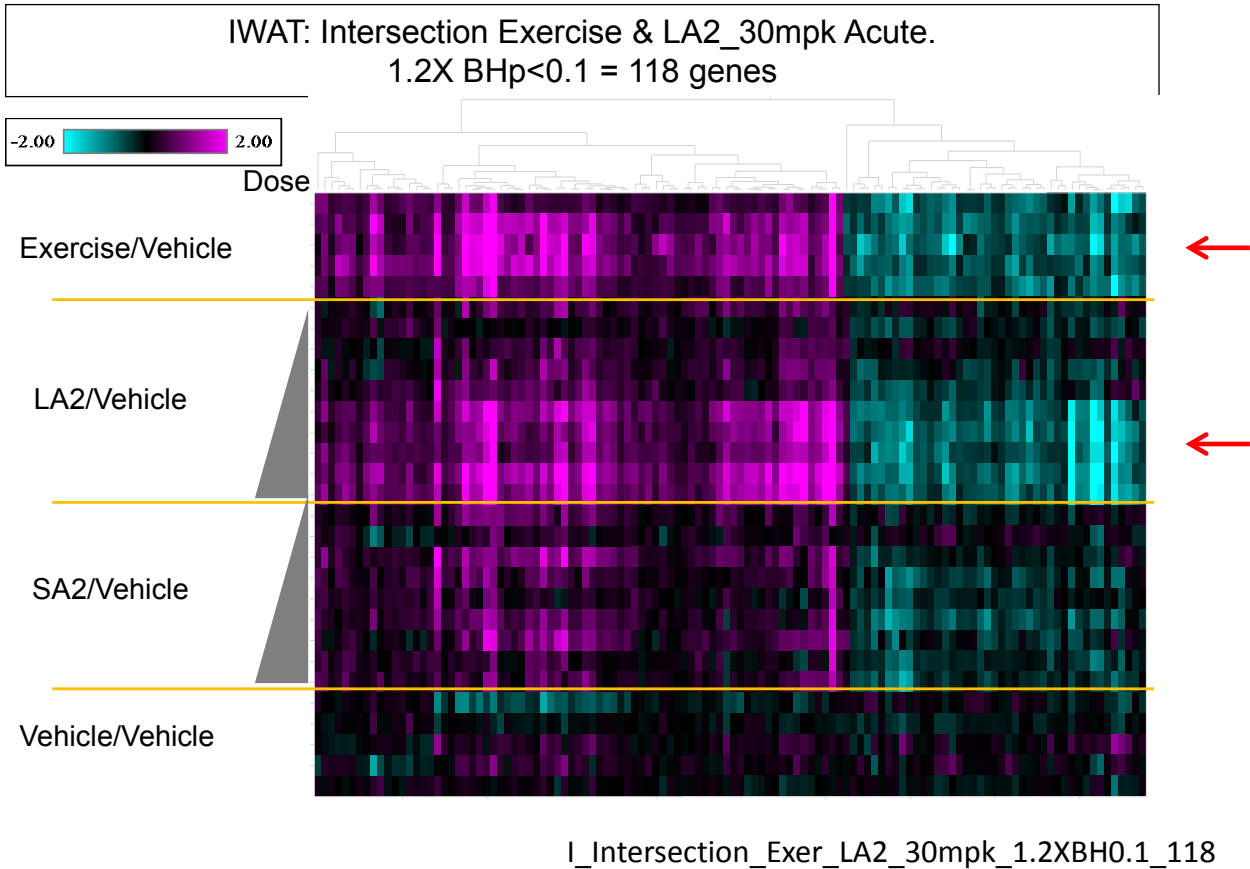

Supplement: S15 Fig — Shown in the heat map are the 118 probesets that met the +/- 1.2 fold change and FDR_BH p<0.1 threshold in the acute exercise group and acute LA2 (high dose) treatment group (red arrows). The color gradient represents fold change compared to vehicle treated sedentary mice (-2.0 to 2.0 fold). The 118 probesets shown here are listed in S17 Table. (PDF) [file pone.0211568.s015.pdf]

S16 Fig.

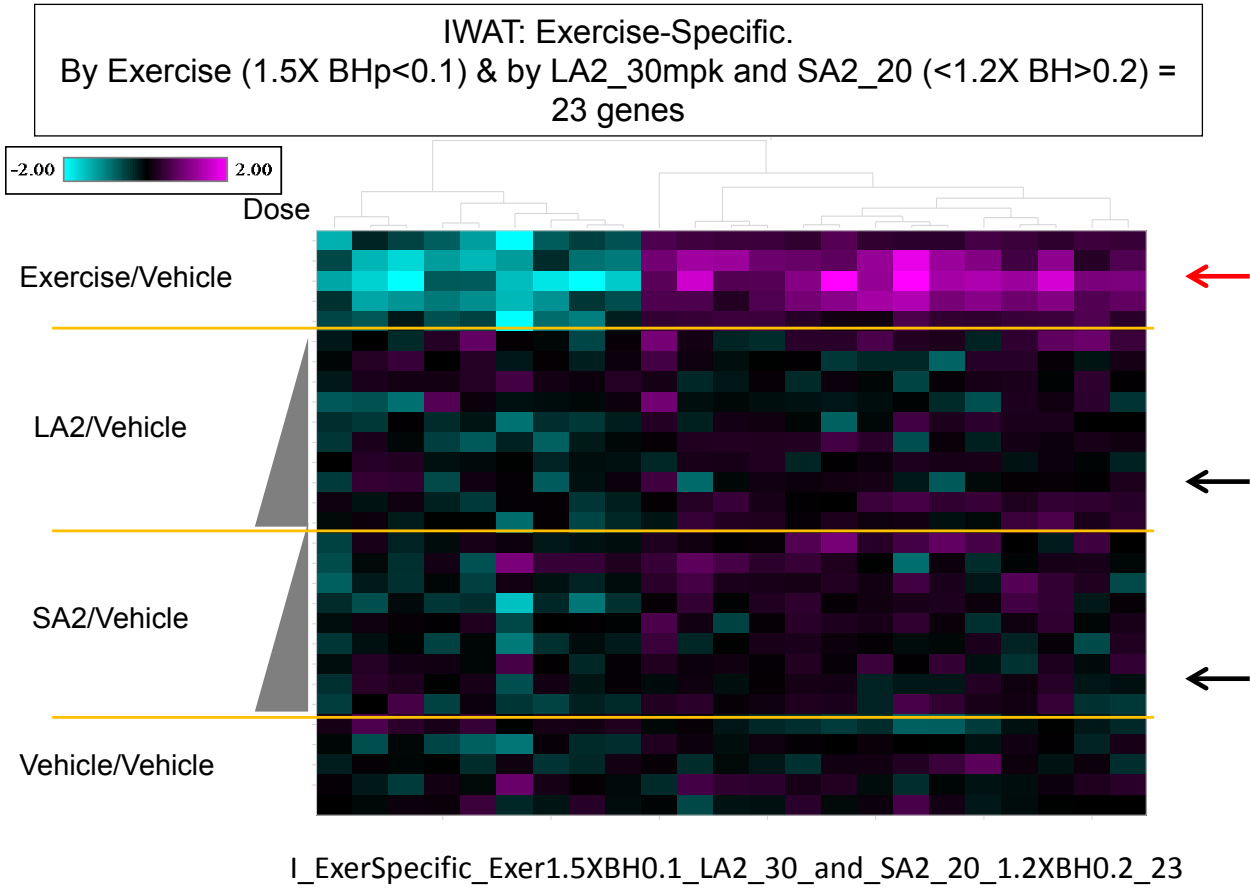

Supplement: S16 Fig — Shown in the heat map are the 23 probesets that met the +/- 1.5 fold change and FDR_BH p<0.1 threshold in the acute exercise group (red arrow), and not significantly changed by LA2 and SA2 treatment (both high dose, and both with < +/- 1.2 fold change and FDR_BH p>0.2; black arrows). The color gradient represents fold change compared to vehicle treated sedentary mice (-2.0 to 2.0 fold). The 23 probesets shown here are listed in S18 Table. (PDF) [file pone.0211568.s016.pdf]

S17 Fig.

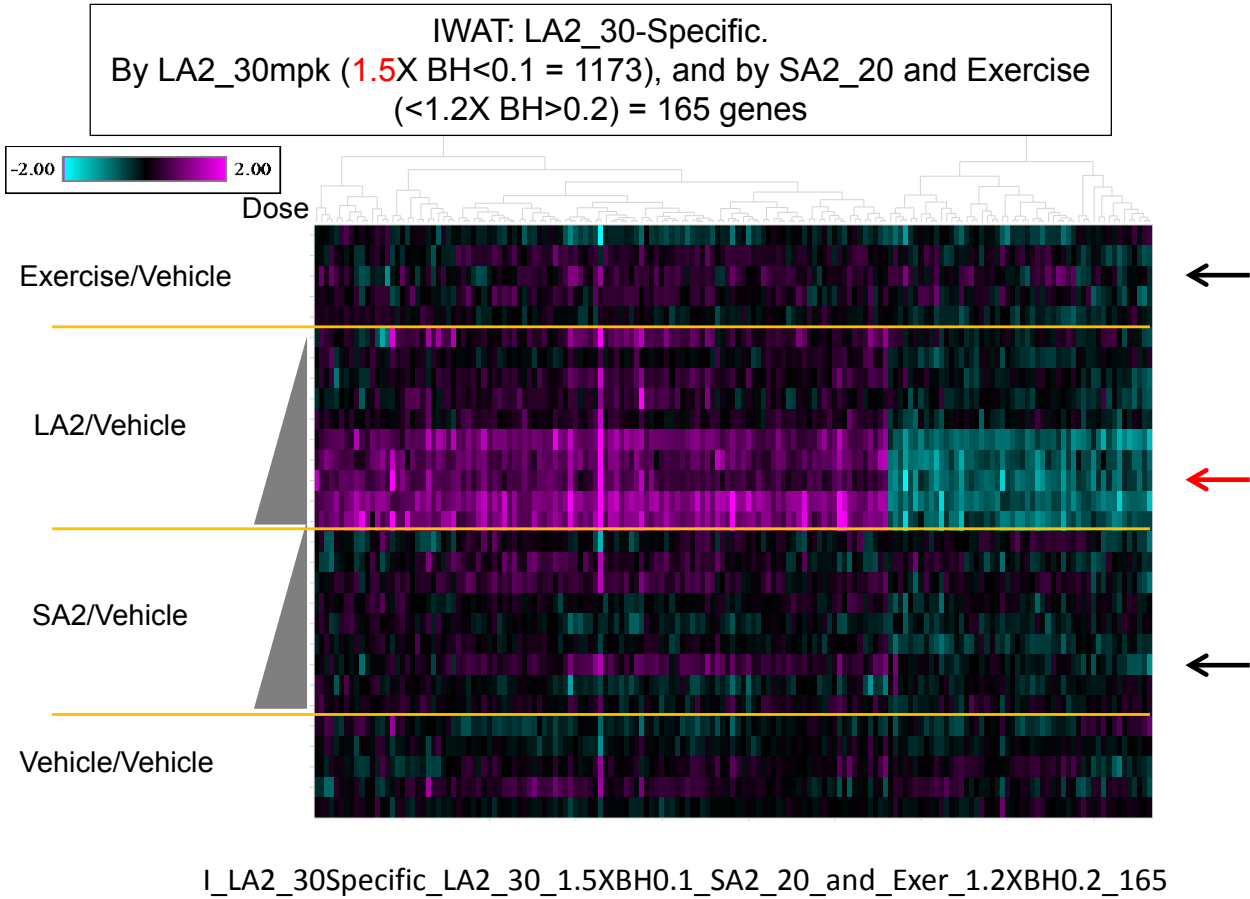

Supplement: S17 Fig — Shown in the heat map are the 165 probesets that were significantly regulated by LA2 (+/- 1.5 fold change and FDR_BH p<0.1; red arrow) and not significantly changed by either SA2 or by acute exercise (< +/- 1.2 fold change and FDR_BH p>0.2) (black arrows).The color gradient represents fold change compared to vehicle treated sedentary mice (-2.0 to 2.0 fold). The 165 probesets shown here are listed in S19 Table. (PDF) [file pone.0211568.s017.pdf]

**S18 Fig.**

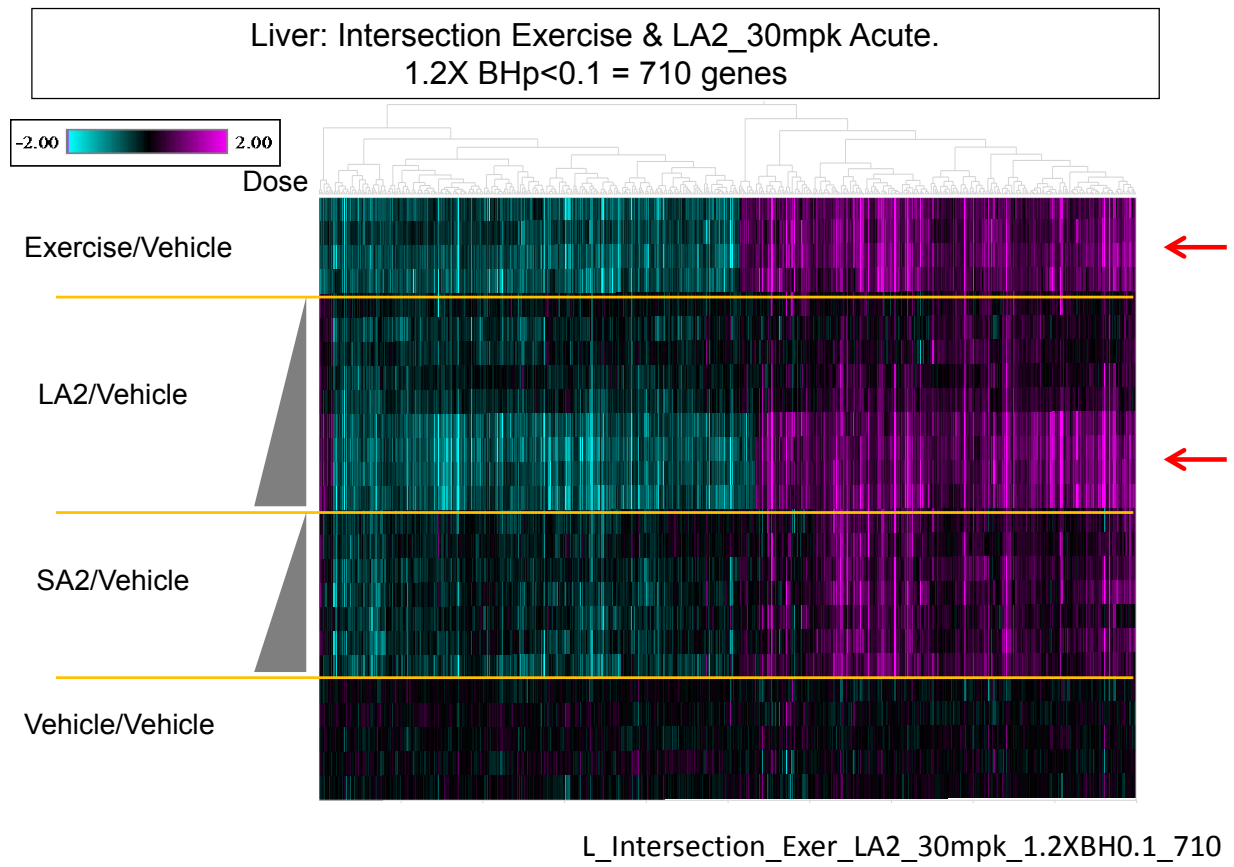

Supplement: S18 Fig — Shown in the heat map are the 710 probesets that met the +/- 1.2 fold change and FDR_BH p<0.1 threshold in the acute exercise group and acute LA2 (high dose) treatment group (red arrows). The color gradient represents fold change compared to vehicle treated sedentary mice (-2.0 to 2.0 fold). The 710 probesets shown here are listed in S20 Table. (PDF) [file pone.0211568.s018.pdf]

S19 Fig.

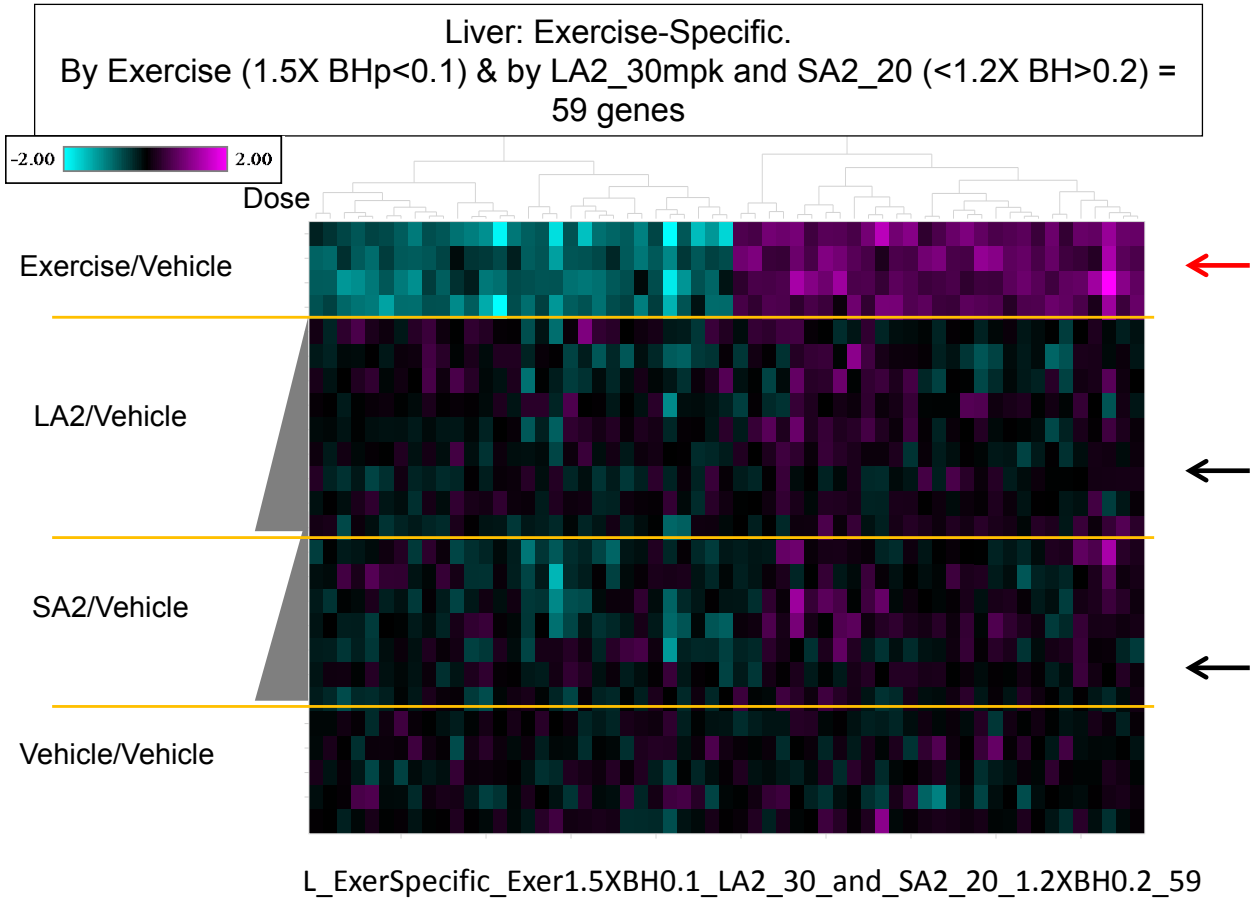

Supplement: S19 Fig — Shown in the heat map are the 59 probesets that met the +/- 1.5 fold change and FDR_BH p<0.1 threshold in the acute exercise group (red arrow), and not significantly changed by LA2 and SA2 treatment (both high dose, and both with < +/- 1.2 fold change and FDR_BH p>0.2; black arrows). The color gradient represents fold change compared to vehicle treated sedentary mice (-2.0 to 2.0 fold). The 59 probesets shown here are listed in S21 Table. (PDF) [file pone.0211568.s019.pdf]

S20 Fig.

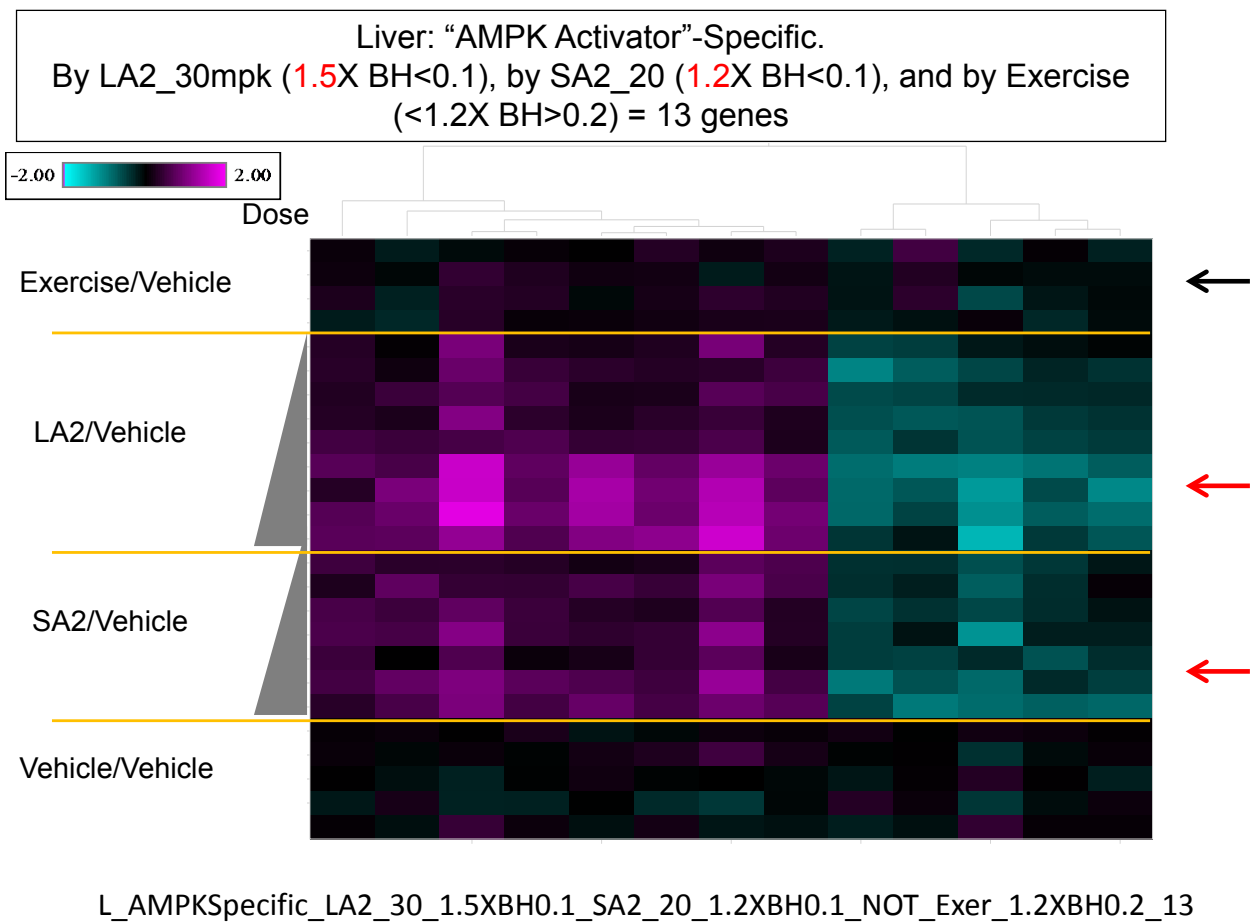

Supplement: S20 Fig — Shown in the heat map are the 13 probesets that were significantly regulated by LA2 (+/- 1.5 fold change and FDR_BH p<0.1) and SA2 (+/- 1.2 fold change and FDR_BH p<0.1) (red arrows), and not significantly changed by acute exercise (< +/- 1.2 fold change and FDR_BH p>0.2; black arrow). The color gradient represents fold change compared to vehicle treated sedentary mice (-2.0 to 2.0 fold). The 13 probesets shown here are listed in S22 Table. (PDF) [file pone.0211568.s020.pdf]

S21 Fig.

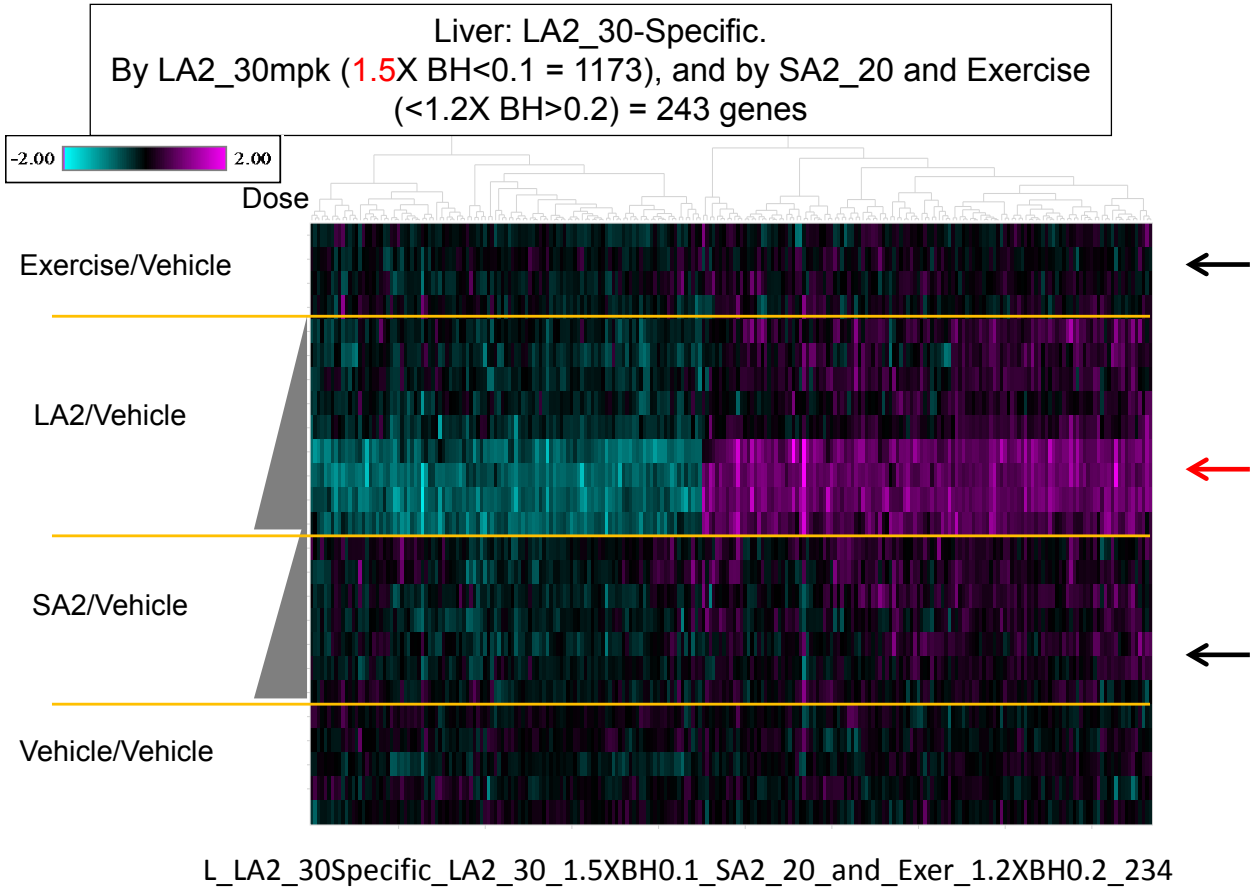

Supplement: S21 Fig — Shown in the heat map are the 243 probesets that were significantly regulated by LA2 (+/- 1.5 fold change and FDR_BH p<0.1; red arrow) and not significantly changed by either SA2 or by acute exercise (< +/- 1.2 fold change and FDR_BH p>0.2) (black arrows).The color gradient represents fold change compared to vehicle treated sedentary mice (-2.0 to 2.0 fold). The 243 probesets shown here are listed in S23 Table. (PDF) [file pone.0211568.s021.pdf]

S22 Fig.

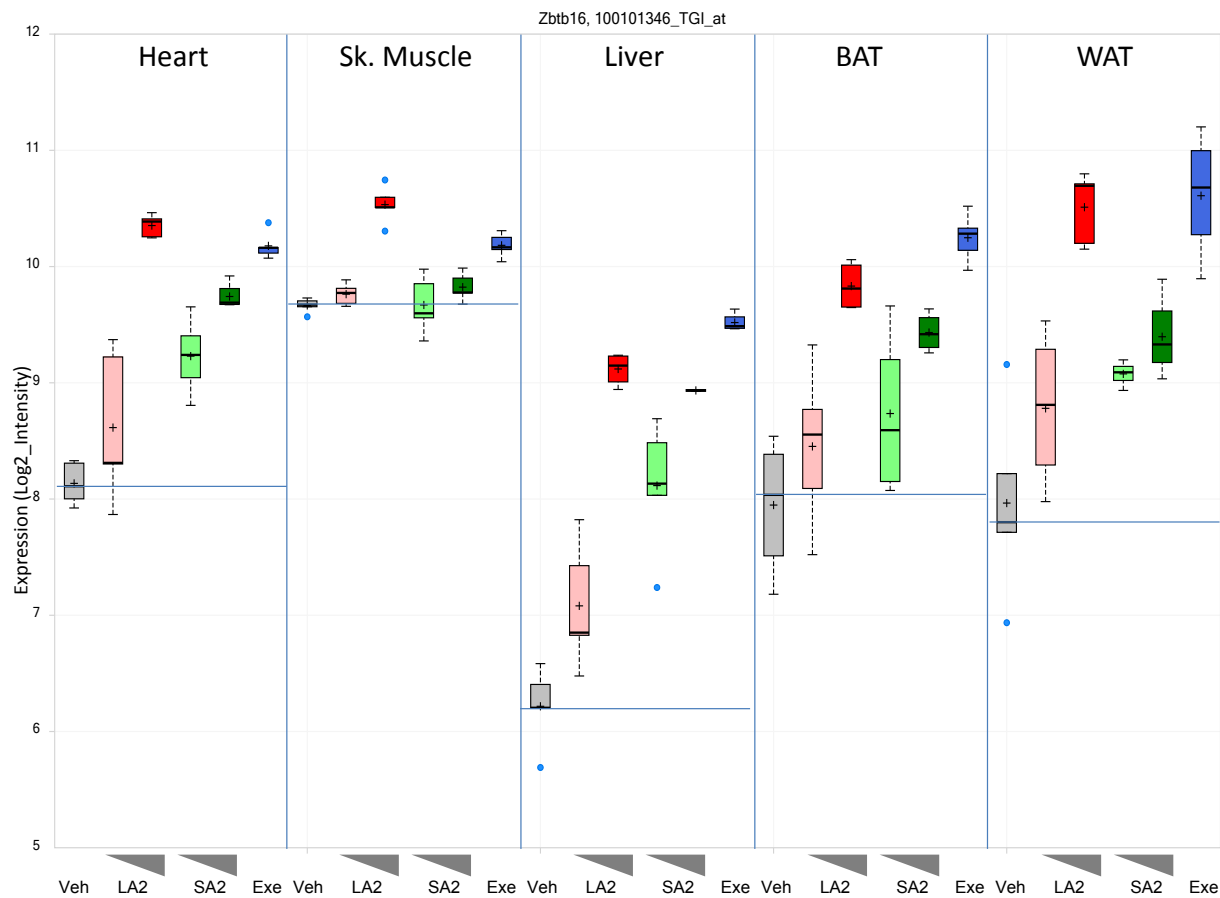

Supplement: S22 Fig — Shown in the box plot are the log2 Intensity values per treatment group. (PDF) [file pone.0211568.s022.pdf]

S23 Fig.

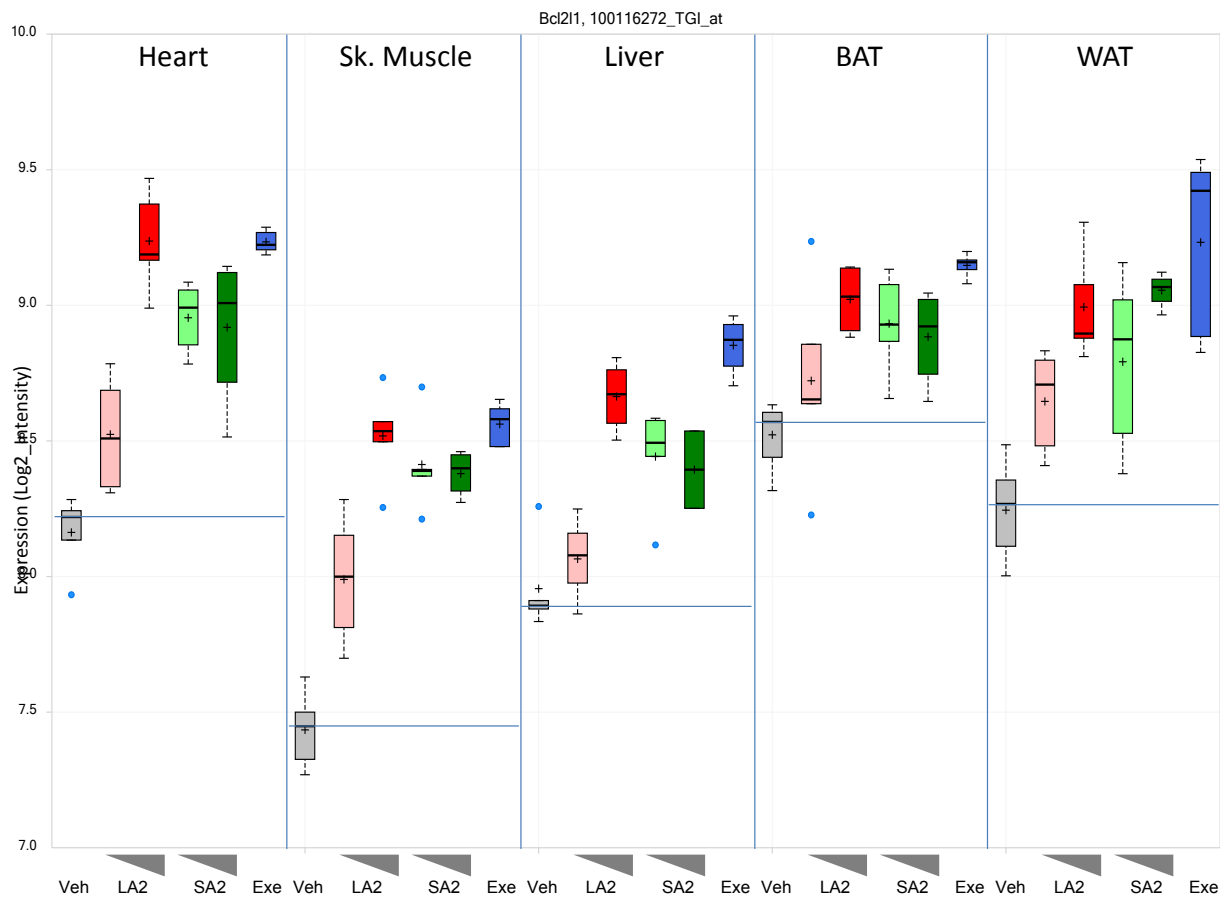

Supplement: S23 Fig — Shown in the box plot are the log2 Intensity values per treatment group. (PDF) [file pone.0211568.s023.pdf]

S24 Fig.

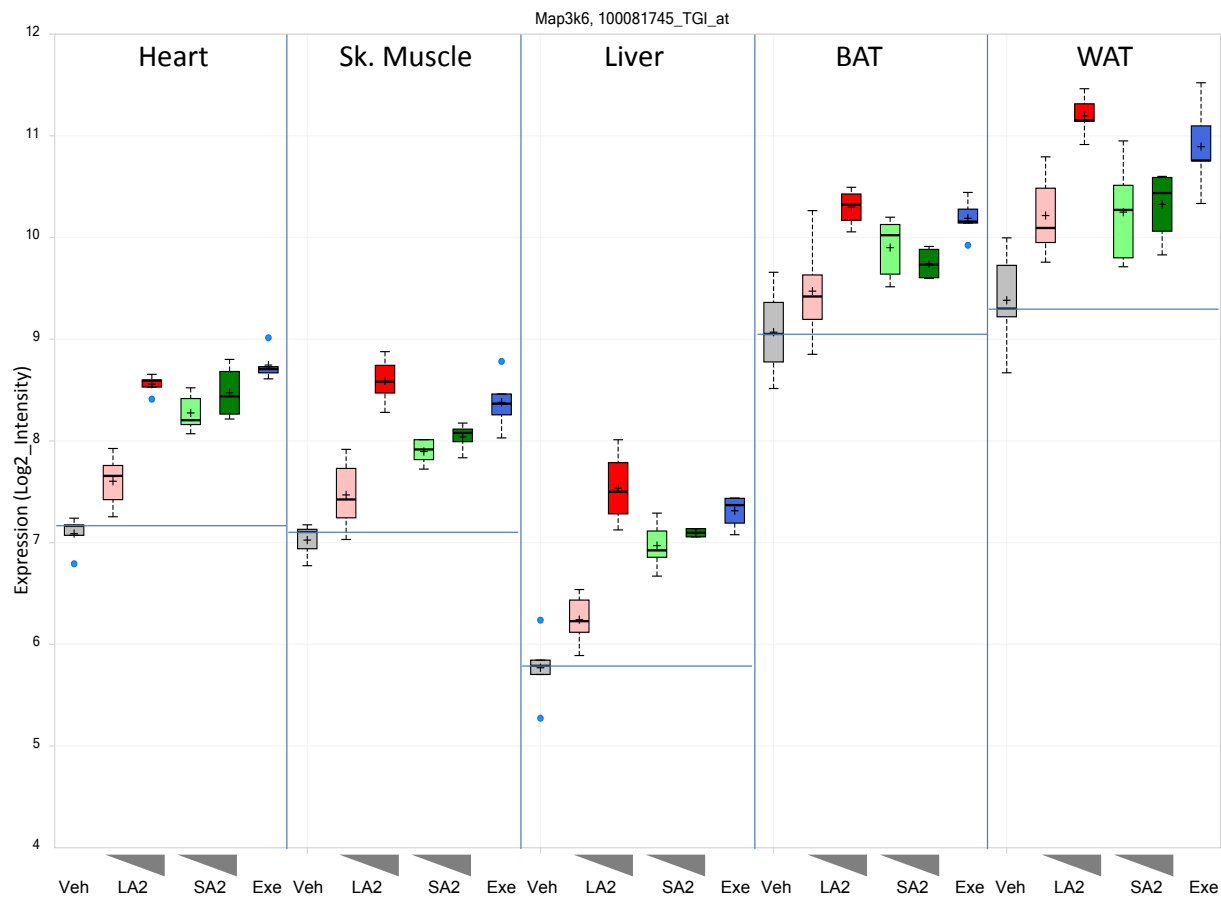

Supplement: S24 Fig — Shown in the box plot are the log2 Intensity values per treatment group. (PDF) [file pone.0211568.s024.pdf]

S25 Fig.

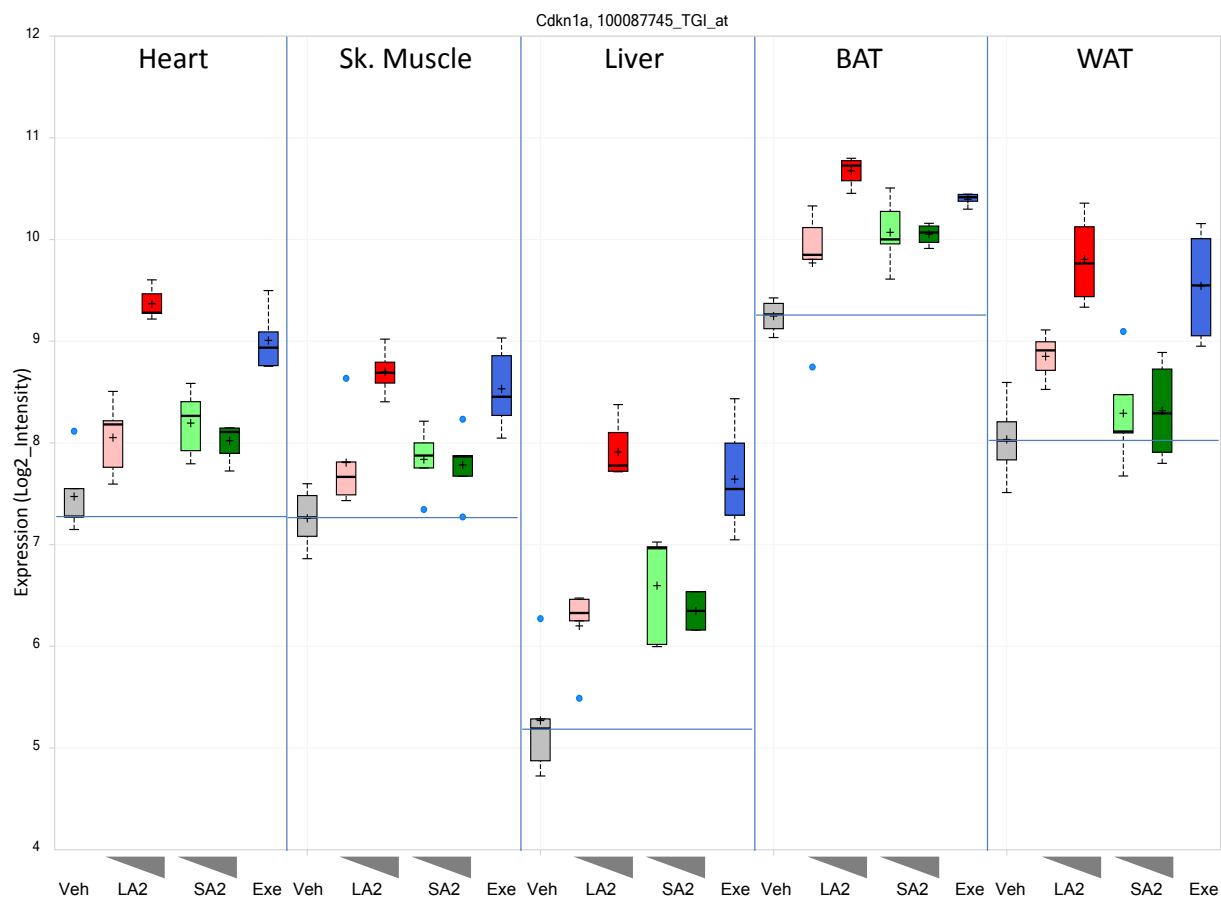

Supplement: S25 Fig — Shown in the box plot are the log2 Intensity values per treatment group. (PDF) [file pone.0211568.s025.pdf]

S26 Fig.

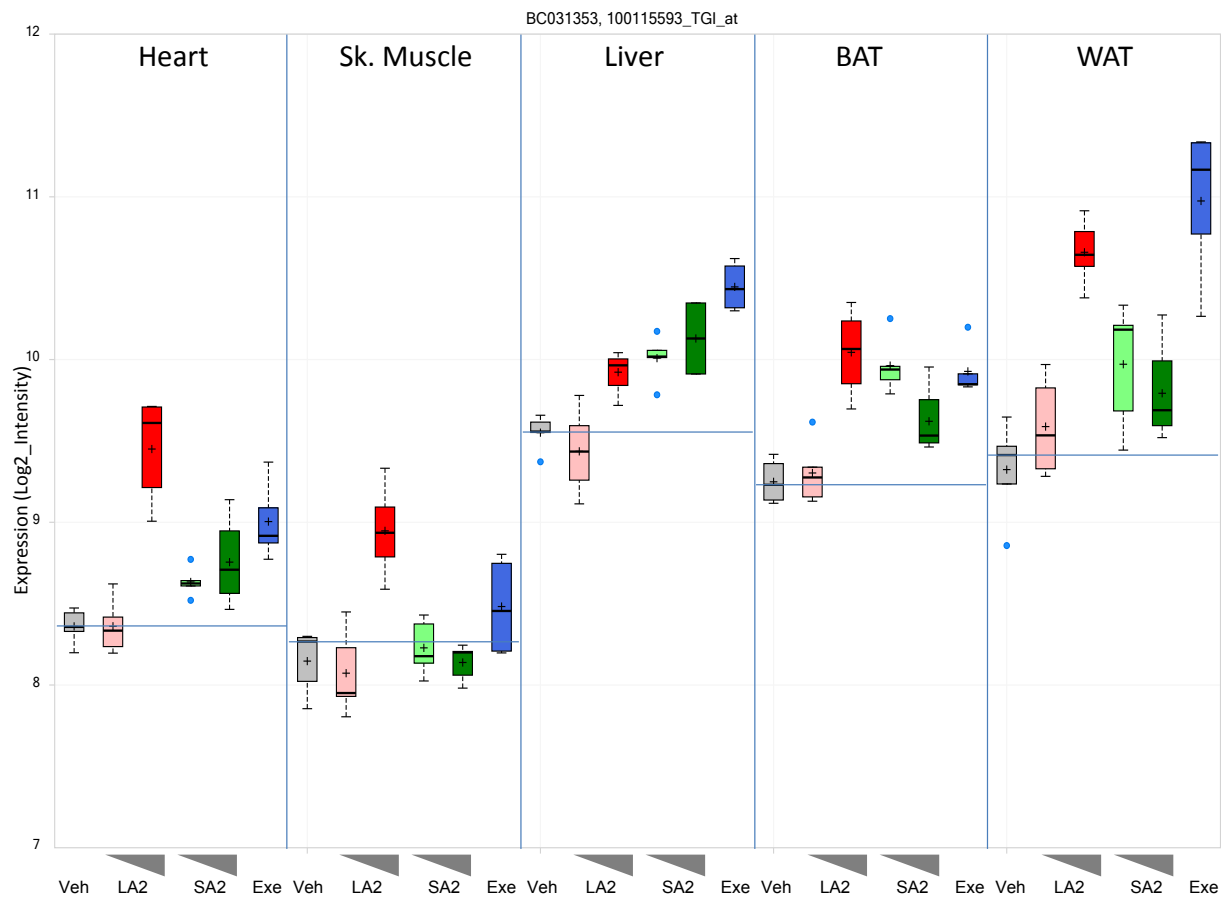

Supplement: S26 Fig — Shown in the box plot are the log2 Intensity values per treatment group. (PDF) [file pone.0211568.s026.pdf]

S27 Fig.

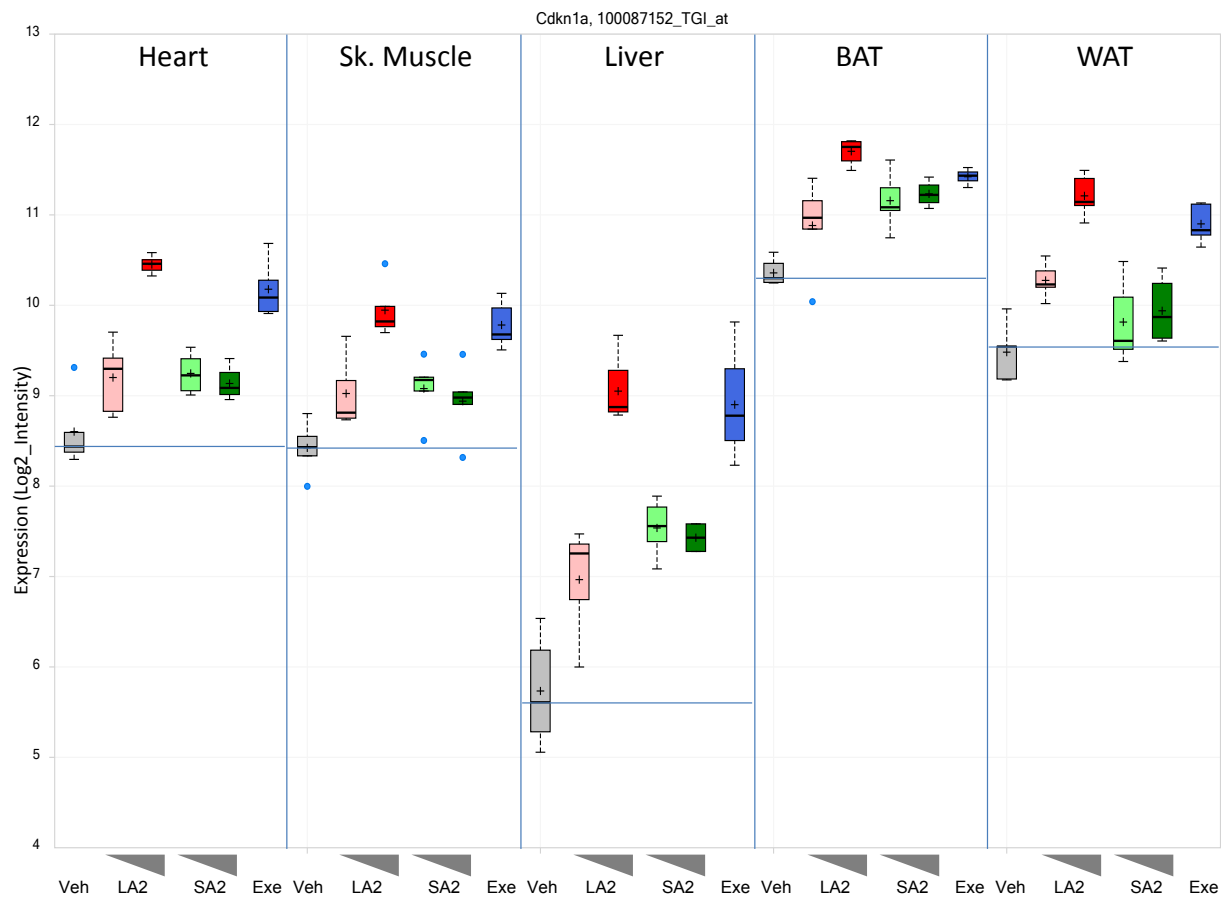

Supplement: S27 Fig — Shown in the box plot are the log2 Intensity values per treatment group. (PDF) [file pone.0211568.s027.pdf]

S28 Fig.

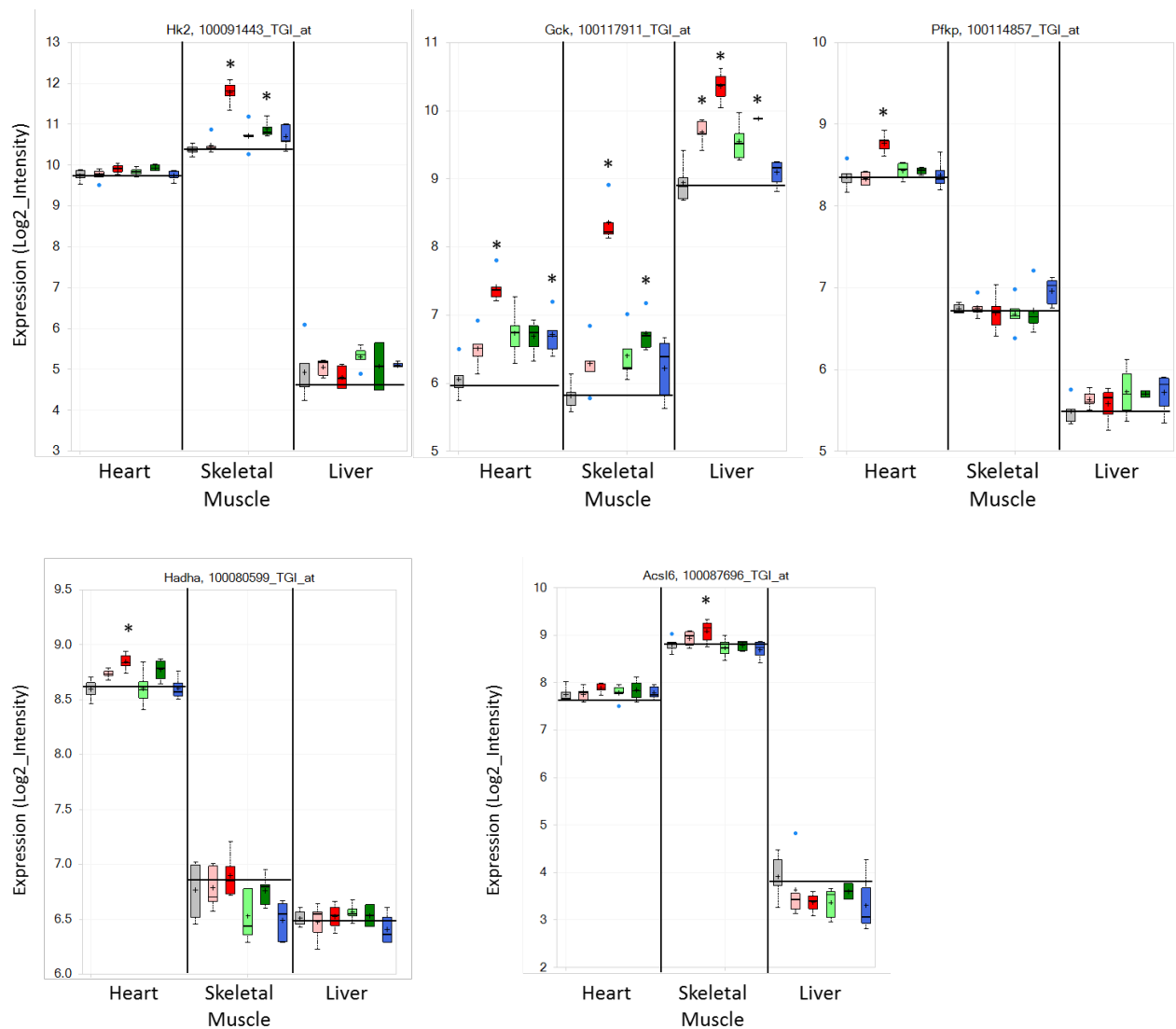

Supplement: S28 Fig — Shown in the box plots are the log2_Intensity values per treatment group for genes involved in glycogen, glucose, and lipid metabolism. See S24 Table for the corresponding data. (PDF) [file pone.0211568.s028.pdf]

S29 Fig.

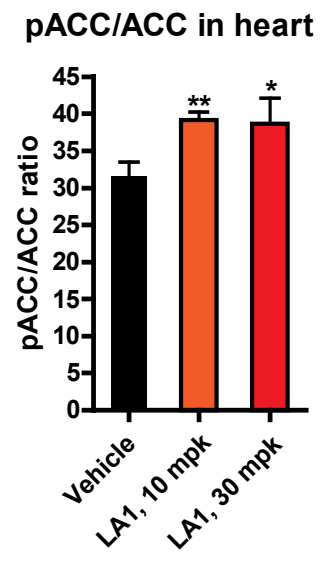

Supplement: S29 Fig — pACC/ACC ratio in the heart of male db/+ mice after 2 hour of treatment of LA1 at 10 and 30 mpk (PO, n = 6). (PDF) [file pone.0211568.s029.pdf]

**S30 Fig.**

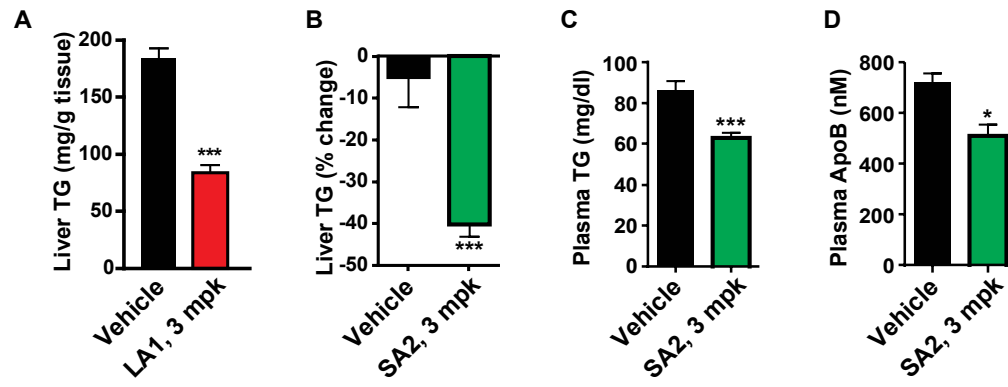

Supplement: S30 Fig — After 12 weeks on high fat diet, the animals were treated by oral administration of LA1 at 3 mg/kg per day for 21 days (A) or SA2 at 3 mpk for 30 days (B–D) (n = 10). Hepatic lipids were measured using magnetic resonance imaging and proton magnetic resonance spectroscopy (1H-MRS) at the end of the study. “Liver TG (% change)” in B is the % change from baseline of the mice after treatment. These mice were imaged prior to treatment, and the treatment effect was calculated based on the TG changes of each individual mouse compared with its own baseline. Plasma triglyceride (TG) and apolipoprotein B (ApoB) were measured by Infinity Triglycerides Reagent (Thermo Scientific) and mouse ApoB ELISA kit (Abcam), respectively. (PDF) [file pone.0211568.s030.pdf]
